# Corporate activities that influence population health: a scoping review and qualitative synthesis to develop the HEALTH-CORP typology

**DOI:** 10.1186/s12992-024-01082-4

**Published:** 2024-11-09

**Authors:** Raquel Burgess, Kate Nyhan, Nicholas Freudenberg, Yusuf Ransome

**Affiliations:** 1 Department of Social and Behavioral Sciences, Yale School of Public Health, 60 College Street, New Haven, CT 06510 United States of America; 2 Harvey Cushing/John Hay Whitney Medical Library, Yale University, 333 Cedar Street, New Haven, CT 06510 United States of America; 3 Department of Environmental Health Sciences, Yale School of Public Health, 60 College Street, New Haven, CT 06510 United States of America; 4 Department of Community Health and Social Sciences, CUNY Graduate School of Public Health & Health Policy, 55 W 125th Street, New York City, 10027 United States of America

## Abstract

**Introduction:**

The concept of the commercial determinants of health (CDH) is used to study the actions of commercial entities and the political and economic systems, structures, and norms that enable these actions and ultimately influence population health and health inequity. The aim of this study was to develop a typology that describes the diverse set of activities through which commercial entities influence population health and health equity across industries.

**Methods:**

We conducted a scoping review to identify articles using CDH terms (*n* = 116) published prior to September 13, 2022 that discuss corporate activities that can influence population health and health equity across 16 industries. We used the qualitative constant comparative method to inductively code descriptions and examples of corporate activities within these articles, arrange the activities into descriptive domains, and generate an overarching typology.

**Results:**

The resulting Corporate Influences on Population Health (HEALTH-CORP) typology identifies 70 corporate activities that can influence health across industries, which are categorized into seven domains of corporate influence (i.e., political practices, preference and perception shaping practices, corporate social responsibility practices, economic practices, products & services, employment practices, and environmental practices). We present a model that situates these domains based on their proximity to health outcomes and identify five population groups (i.e., consumers, workers, disadvantaged groups, vulnerable groups, and local communities) to consider when evaluating corporate health impacts.

**Discussion:**

The HEALTH-CORP typology facilitates an understanding of the diverse set of corporate activities that can influence population health and the population groups affected by these activities. We discuss how the HEALTH-CORP model and typology could be used to support the work of policy makers and civil society actors, as well as provide the conceptual infrastructure for future surveillance efforts to monitor corporate practices that affect health across industries. Finally, we discuss two gaps in the CDH literature that we identified based on our findings: the lack of research on environmental and employment practices and a dearth of scholarship dedicated to investigating corporate practices in low- and middle-income countries. We propose potential avenues to address these gaps (e.g., aligning CDH monitoring with other occupational health monitoring initiatives).

**Supplementary Information:**

The online version contains supplementary material available at 10.1186/s12992-024-01082-4.

## Introduction

For centuries, commercial actors and the structures that govern them have shaped the health of various populations in profound ways [1]. Growing attention to this influence recognizes the increasing economic, political, and legal power wielded by commercial entities, especially those that operate transnationally. In the past two decades, scholars have studied this issue through the lens of the ‘commercial determinants of health’ (CDH). In a recent *Lancet*-commissioned series on the topic, the CDH were defined as “the systems, practices, and pathways through which commercial actors drive health and equity” [2].

Research undertaken to understand the nature of these systems, practices, and pathways has proliferated in the last ten years [3]. In 2015, Baum and colleagues [4] proposed a framework to guide assessments of the health impact of transnational corporations; they suggested evaluating the company’s structure (e.g., supply chain) and practices (e.g., political practices). The research team later applied this approach to investigating the health impacts of *McDonald’s Australia* and *Rio Tinto* (an extractive company) [5, 6]. In 2018, Madureira Lima and Galea published a seminal framework describing five ‘vehicles of power’ (e.g., knowledge environment) through which commercial entities shape population health [7]. They suggested that commercial actors engage in practices (e.g., funding medical education) that allow them to exert power through these vehicles, ultimately leading to adverse health outcomes. Several other useful frameworks have been developed to explain the influence of commercial entities on population health. These frameworks provide detailed descriptions of specific types of corporate practices (e.g., corporate political activity) [8–12] and/or describe the activities of particular industries that impact population health (e.g., the food & beverage industry) [8–10, 13]. Other proposed models depict how concepts such as power and approaches such as systems thinking can be used to further illuminate the influence that commercial entities have on human health [14, 15].

The most comprehensive model of the influence of commercial entities on population health to-date was published in the 2023 *Lancet* series on the CDH. Building on existing models, Gilmore and colleagues [2] proposed a model that describes how the growth strategies and business models of commercial entities determine their engagement in seven types of practices (e.g., political practices, labour and employment practices). The authors proposed that companies’ engagement in these practices exerts influence on the political and economic system, which has downstream impacts on social determinants of health (SDH) such as housing. Lacy-Nichols and colleagues [16] further expanded on this model by identifying four attributes (i.e., portfolio, resources, organization, and transparency) that can be used to both differentiate between commercial entities and explain and predict their engagement in certain practices. The authors provide a set of guiding questions (e.g., “What is the nature of the entity’s employment contracts?”) across these seven practices and four attributes that can be used by various stakeholders (e.g., policy makers) to assess the risks of engaging with companies on public health issues and guide efforts to research and monitor commercial entities and their practices.

While the 2023 *Lancet* series identifies seven different types of commercial practices (comprehensive approach) and previous typologies provide us with in-depth descriptions of specific practices (detailed approach), what is missing from the literature is a framework that provides a comprehensive, yet detailed overview of the diverse set of activities through which commercial entities can influence population health and health equity across industries. This type of framework could be used as a resource for policy makers, civil society actors, and others (e.g., investors) to consider the actions of commercial entities and their implications for population health. It could also provide the conceptual infrastructure for future efforts to monitor commercial practices across industries.

To address this gap, we conducted a systematic scoping review and qualitative synthesis of CDH articles that discussed corporate activities that have the potential to influence population health and health equity. Leveraging the findings from this process, we developed a typology (called the Corporate Influences on Population Health (HEALTH-CORP) typology) that identifies 70 specific corporate activities with the potential to influence human health across industries and categorizes these activities into seven domains of corporate influence (e.g., products and services, employment practices). We propose a model that positions these domains based on their proximity to health outcomes (i.e., distal to proximal) and identifies five population groups (e.g., consumers, local communities) to consider when evaluating the health impact of corporate practices. Our final contribution is to leverage the findings to illuminate current gaps in CDH research.

In the following sections, we report our review methods and describe how we used the literature to build the HEALTH-CORP typology. We summarize the activities that comprise the seven domains of corporate influence and those identified as influencing the health of the five population groups. We conclude by discussing the utility of these contributions and identifying avenues for future CDH research.

### Methods

We used scoping review methodology to find relevant literature and then qualitatively synthesized this literature to build a typology. We chose qualitative synthesis as our analytical method as it is useful for systematically interpreting research to identify and represent its meaning [17]. We also performed a separate descriptive analysis of the characteristics of the same literature (e.g., industries and regions investigated, methods used); the results of this analysis are published elsewhere [3]. Our methods are summarized below; a fuller description of our search, screening, and data extraction procedures can be found in our complementary article [3].

### Search strategy

We conducted the search originally developed by de Lacy-Vawdon and Livingstone [18], which we expanded to include additional CDH-related terms (e.g., corporate political activity) (Appendix 1). We performed the search on January 4, 2022 and again on September 13, 2022 with no date restrictions.

The title, abstract, and full text screening was conducted by R.B. and we did not employ double, independent screening for verification purposes. During full-text screening, articles that did not mention CDH terms (i.e., “commercial determinants”, “corporate determinant(s)”) were excluded. The purpose of this strategy was to identify as many CDH articles as possible, including those that mentioned CDH terms in the full text but not within the title, abstract, or keywords.

### Eligibility criteria

Eligible articles were written in the English language and described activities (i.e., decisions, strategies, or other actions) that corporations or those acting on behalf of corporations engage in that have been demonstrated to or have the potential to influence population health and/or health equity. This criterion was applied broadly to identify as many relevant activities as possible; the potential to influence population health and/or health equity could have been investigated explicitly within the respective study, supported by previous research (for e.g., changes to income are known to influence health), or reasonably be expected to influence population health (e.g., delayed implementation of evidence-based health policy due to corporate influence). Moreover, the expected impact on health could be direct (e.g., occupational injury), indirect (e.g., decreased health protections due to corporate lobbying), health-promoting (e.g., provision of income), or health-adverse (e.g., harmful product), or some combination of these categories. Consistent with scoping review methodology [19], a formal quality assessment of the methods reported in the included articles was not performed.

### Data extraction

We extracted information about the characteristics of the included articles: the year of publication, methodology employed (e.g., qualitative), industry/ies discussed (e.g., alcohol), region(s) studied (e.g., Brazil), reported funding status (yes; no), type of funder (e.g., government entity), reported conflicts of interest (yes; no), the journal in which the article was published, and the article’s open-access status (yes; no) [3]. The findings from this data are published elsewhere [3].

### Data synthesis and development of HEALTH-CORP typology and model

Using the qualitative data analysis software NVivo 12 [20], the first author (R.B.) employed the constant comparative method [21] to inductively code the data with the goal of identifying relevant corporate activities and arranging them into overarching descriptive domains. The constant comparative method involves coding ‘incidents’ (i.e., examples/cases) within the data to larger categories of analysis, comparing incidents within categories, and then integrating these categories to identify the properties of a phenomenon of interest [21, 22]. In this case, examples of corporate activities discussed in the included texts (e.g., corporate involvement in nutrition education in schools [23]) were coded and compared to other relevant examples (e.g., corporate provision of resources on breastfeeding for mothers [24]), to identify overarching activities (e.g., corporate involvement in health education directed at the public). These activities were then grouped into larger descriptive categories (e.g., preference and perception shaping practices), which we call ‘domains of corporate influence’. Specific activities were included in the typology if they had been described in a general sense in the included literature (i.e., without reference to any specific industry) or discussed in relation to two or more industries; see Appendix 5 for a list of activities described in reference to one industry. Appendix 6 shows which domains were discussed in relation to each industry.

Next, the first author identified five population groups whose health is affected by corporate activities (i.e., consumers, workers, disadvantaged groups, vulnerable groups, and local communities). These groups were identified based on the findings from the qualitative analysis, which revealed examples of corporate activities (e.g., child labour) that affected specific groups (i.e., children, a vulnerable group). The population groups were further refined by consulting the frameworks developed by the Access to Nutrition Initiative (ATNI) and ShareAction. ATNI assesses food and beverage manufacturers in terms of their contributions to the nutrition of priority populations (i.e., those at-risk due to life stage, age, income, culture, or physical access) [25]. ShareAction supports investors in prioritizing health by considering the impacts of companies on the health of workers, consumers, and communities [26]. After identifying the five population groups, R.B. reviewed the initial analysis to gain insight into the domains and activities that can influence their health (see ‘Health Impact on Populations’ in Results).

Finally, the first author (R.B.) developed a model to depict the relationships between the domains of corporate influence and the health of the identified population groups. This model was developed by reviewing the findings from the qualitative analysis and by consulting and drawing from other models of corporate influence on population health [2, 4, 7, 11, 13–16, 27–29].

Though the analysis and typology development was led by the first author (R.B.), the third (N.F.) and final author (Y.R.) were consulted to provide input on the structure and clarity of the resulting HEALTH-CORP model. Moreover, following the development of the HEALTH-CORP typology, our author team conducted a follow-up study to adapt the typology to the food and beverage industry (forthcoming). In the context of this study, we received comments on the clarity and structure of the HEALTH-CORP typology from 22 public health professionals and academics, including CDH scholars. These comments were used to further refine the HEALTH-CORP typology.

## Results 

### Characteristics of articles included in the review

Following the screening process, a total of 116 articles were included in the analysis (see Appendix 2 for the PRISMA diagram) [3]. Almost half of the articles included were conceptual (50 articles; 43%) and a substantial proportion employed qualitative methods (37; 32%). The articles described corporate activities that were undertaken by 16 different industries: food & beverage, tobacco, alcohol, baby food, gambling, pharmaceuticals and diagnostics, extractive, firearms, social media, cannabis, fossil fuels, e-cigarettes/vape, prisons, retail, crowdfunding, and housing. Fifty eight percent (67 articles) focused on the food and beverage, tobacco, and/or alcohol industries, with less research dedicated to studying other industries. Of the 58 included articles that reported a regional focus, 72% (42 articles) studied corporate activities in high-income countries (HICs), such as United States, United Kingdom, or Australia. A detailed analysis of the characteristics of the included literature is reported in our previous work [3]. Individual article characteristics are provided in Appendix 3.

### Corporate influences on population health (HEALTH-CORP)

We identified seven domains of corporate influence, which are: 1) political practices, 2) preference and perception shaping practices, 3) corporate social responsibility practices, 4) economic practices, 5) products and services, 6) employment practices, and 7) environmental practices (Table 1).

**Table 1:** Definitions of Domains of Corporate Influence

**Domains of Corporate Influence**	**Definition**
Political Practices	This domain consists of activities undertaken to influence government policy or processes in ways that are favourable to the commercial entity [7].

Preference & Perception Shaping Practices	This domain consists of activities that shape preferences for products and/or influence perceptions about products and their health-related harms [7].

Corporate Social Responsibility Practices	This domain consists of activities undertaken with the stated intention to contribute to society and/or offset environmental, social, or health impacts of previous activities [34].

Economic Practices	This domain consists of activities that influence the economy and the distribution of wealth within and across societies [4].

Products & Services	This domain consists of activities related to the production and sale of products and services [35].

Employment Practices	This domain consists of activities related to the conditions under which employment is provided [36].

Environmental Practices	This domain consists of activities that can influence physical and/or mental health through the impact of the activity on the natural environment [37].

**Fig. 1:**
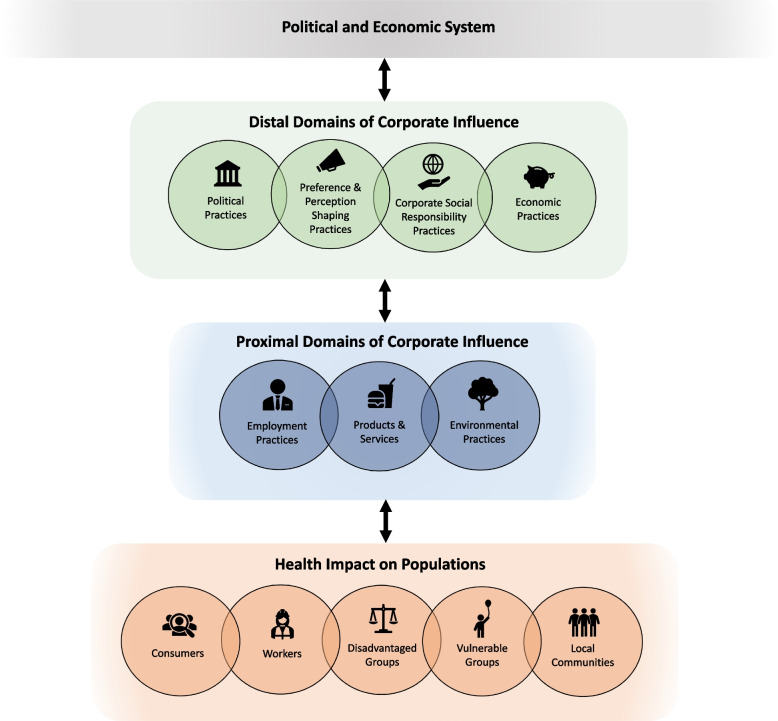
The Corporate Influences on Population Health (HEALTH-CORP) Model

In Fig. 1, we provide a graphical depiction of the domains. Similar to Gilmore and colleagues [2], we use overlapping circles to demonstrate that the domains are not mutually exclusive and can be interactive. For example, some activities (e.g., ‘Conduct educational and/or advocacy campaigns to influence the public's perception of health policies’) contain elements of both preference and perception shaping practices and political strategies (not mutually exclusive). Likewise, activities such as ‘obscure conflicts of interest in research’ could influence other activities such as the ‘Misrepresent evidence or demand unrealistic standards of evidence  within policy submission processes’ (interactive).

Political practices, preference and perception shaping practices, corporate social responsibility practices, and economic practices are positioned as distal domains (i.e., their impact on population health is indirect). Employment practices, products and services, and environmental practices are positioned as proximal domains (i.e., their impact on population health is direct) [30]. A vertical double-headed arrow between the proximal and distal domains suggest that the distal domains enable the proximal domains and the proximal domains reinforce the distal domains. That is, by shaping the political, normative, epistemic, and economic environment in favourable ways, corporations are able to influence the conditions in which they operate (e.g., the environmental impacts for which they are held accountable) [2]. In turn, the proximal domains can simultaneously influence the extent to which commercial entities can engage in the distal domains. For example, transnational companies can leverage their employment of large numbers of people (employment practices) to impose pressure on political and judicial processes (political practices), as was the case with the SNC-Lavalin scandal in Canada in 2019 [31]. Similarly, pharmaceutical companies in the United States were able to leverage their production of necessary products to advocate for reduced liability for product-related harms [32].

The domains of corporate influence are depicted as influencing the health of five different population groups: consumers, workers, disadvantaged groups, vulnerable groups, and local communities located near a production or processing facility (Table 2). Though the health of these groups is affected by corporate practices, the double headed arrow portrays that they also have agency to shape corporate practices. For example, workers’ unions, environmental groups, and consumer rights groups can pressure or encourage corporations to change their practices via mechanisms such as strikes, boycotts, stakeholder engagement, shareholder activism, and litigation [1, 33].

**Table 2:** The Corporate Influences on Population Health (HEALTH-CORP) Typology, consisting of seven domains of corporate influence and 70 activities through which commercial entities can influence population health and/or health equity

**Type of Domain** (i.e., Distal or Proximal)	**Domains of Corporate Influence**	**Corporate Activities with Potential to Influence Population Health and/or Health Equity**^a^
**Distal Domains**	**Political Practices**	*Activities related to securing a favourable policy environment: *
		Engage in political financing
		Engage in bribery
		Advocate for policies that limit corporate liability for health harms
		Build relationships with public health institutions and/or other relevant groups (e.g., patient groups)
		Advocate for the placement of corporate representatives on relevant associations and boards
		Exploit the use of revolving doors (i.e., employees who move between positions in industry and government)
		Take or threaten legal action in response to unfavourable policies
		Advocate for self-regulation, co-regulation, or voluntary codes
		Engage in lobbying (including via third parties such as front groups)
		Engage in strategies to leverage pre-emption or venue-shifting (i.e., transfer of policy making towards jurisdictions that are more likely to advance the company’s interests)
		­ Leverage trade and investment treaties to challenge unfavourable policies
		Amplify influence via front groups and/or industry alliances
		Use argumentative strategies within policy submission processes to oppose and/or delay unfavourable policies
		Misrepresent evidence or demand unrealistic standards of evidence within policy submission processes
		Exploit divisions in the public health community
		Intimidate and/or discredit opponents
		Shift or threaten to shift operations to regions with weaker regulations
		*Other political activities:*
		Advocate for privatization of public services
		Expropriate land for industry activities
	**Preference and Perception Shaping Practices**	*Activities related to promoting products: *
		Engage in marketing to encourage product recognition and consumption
		Engage in marketing of harmful products in ways that disproportionately target disadvantaged or vulnerable groups
		Leverage pandemics or disasters to promote consumption of harmful products
		Sponsor sports, music, and cultural events, individuals (e.g., athletes), and/or related infrastructure (e.g., arenas)
		*Activities related to shaping the public debate about products & their health implications: *
		Engage in health education efforts directed at the public
		Conduct educational and/or advocacy campaigns to influence the public’s perception of health policies
		Craft and/or propagate inaccurate or skewed narratives about the causes of health & disease
		Advance the ideas of individual responsibility and consumer choice
		Acquire ownership, establish relationships, or exert influence on the media via advertising spending
		Employ experts and key opinion leaders to further industry-friendly narratives (e.g., by writing position papers)
		*Activities related to shaping the professional debate about human health and nutrition: *
		Fund professional associations
		Contribute to the development of clinical standards
		Engage in health education efforts directed at health care providers or health professions students
		*Activities related to shaping the production and interpretation of evidence: *
		Fund external stakeholders to conduct and/or disseminate research in ways that can shape both the direction (i.e., the topic investigated) and the outcomes (i.e., findings and conclusions) of the research
		Suppress, amplify, or cherry-pick evidence or attempt to generate uncertainty
		Obscure conflicts of interest in research
		Contribute to the development of scientific standards
		Falsify or misrepresent data
	**Corporate Social Responsibility (CSR) Practices**	Develop or contribute to health promotion programs and health-related charities or non-profit organizations
		Develop or contribute to other social responsibility efforts relevant to health (e.g., diversity initiatives)
		Engage with existing social movements (e.g., women’s rights)
		Engage in product reformulation
	**Economic Practices**	Engage in fair or unfair tax practices
		Engage in price fixing or profiteering
		Contribute to economic growth and related benefits (e.g., improvements in infrastructure, education, healthcare)
		Contribute to inequitable distributions of wealth and power (e.g., through ownership and remuneration structures)
**Proximal Domains**	**Products and Services**	*Activities related to the characteristics of products: *
		Develop, produce, or sell products with harmful or salutogenic properties or those that are essential for human life (e.g., food)
		Develop, produce, or sell products with addictive properties
		*Activities related to the accessibility of products: *
		Determine the price of products and implement price promotions, discounts, coupons, offers, and vouchers
		Determine the physical proximity and availability of products to consumers
	**Employment Practices**	*Activities related to determining the characteristics of employment:*
		Determine the number of employment opportunities, their type (e.g., skill level), and geographic distribution
		Determine the adequacy of pay in relation to local living standards
		Determine the stability of employment terms
		*Activities related to the benefits received through employment: *
		Determine the provision and quality of medical benefits
		Determine the provision and quality of pension plans
		Determine the provision, length, and paid or unpaid status of employee leave (e.g., parental, personal)
		Determine the provision and quality of employee wellness programs
		*Activities related to the conditions of employment: *
		Determine the quality of working conditions (physical and psychosocial)
		Determine the extent to which workers are free to engage in unionization or collective bargaining without interference or fear of reprisal
		Determine the extent of support for breastfeeding in the workplace
		Determine the provision of opportunities to work remotely and characteristics of remote work
		*Other employment-related activities: *
		Determine the use of child or forced labour directly or in the supply chain
	**Environmental Practices**	Use (or avoid the use of) harmful chemicals and pesticides
		Determine extent of contributions to air pollution, including efforts to prevent pollution
		Determine extent of contributions to water pollution, including efforts to prevent pollution
		Determine the extent of waste production, including efforts to reduce, reuse, and recycle
		Determine the extent of resource extraction (e.g., water), including efforts to conserve resources
		Determine contributions to deforestation, including efforts to prevent deforestation and reforest
		Determine the extent and source (e.g., renewable) of energy consumption, including efforts to conserve energy
		Determine the extent of greenhouse gas emissions, including efforts to reduce emissions
		Determine the extent of contributions to the loss of biodiversity, including efforts to prevent loss and engage in ecosystem restoration

^a^Though we have focused on corporate agency in this article, many of these activities (e.g., parental leave benefits) are influenced by the actions of other actors (e.g., government) and not solely determined by the company

Finally, though this was not the focus of our study, the political and economic system is depicted above the domains of corporate influence to acknowledge that the identified domains and population groups exist within the context of this larger system. This system has an array of features (e.g., norms, values), structures (e.g., forms of business organization), and actors (e.g., think tanks, government agencies, consultancy firms) that enable and constrain the extent to which commercial entities engage in these practices. Commercial entities can similarly influence this system in ways that are favourable to their objectives [2, 16].

In the next section, we describe the activities that comprise each domain and report the ways these activities were discussed in the included literature. The number of references pertaining to a specific activity should not be interpreted as an indication of the strength of associated evidence, but rather the number of instances the respective activity was discussed in the included articles. Our descriptions of the activities in the political and preference and perception shaping practices domains are brief as these practices have been discussed in detail elsewhere [7, 8, 11, 13]. The HEALTH-CORP typology is presented in Table 2 and an expanded version with additional details is provided in Appendix 4. Following this, we provide descriptions of the domains that influence the health of the five identified populations (see ‘Health Impact on Populations’).

### Distal Domains of Corporate Influence

#### Political practices

Corporate involvement in the development and implementation of health policy was a significant focus of many of the included articles and a wide range of corporate strategies were discussed. Corporations were reported to engage in efforts to secure a favourable policy environment, for instance, by making financial contributions to political parties [18, 22, 24, 38–50] and developing relationships with public health institutions [24, 34, 39, 44, 51–57]. For example, Maani and colleagues [52] used Freedom of Information Act Requests to access emails between *the Coca Cola Company* and the *Centers for Disease Control and Prevention* (CDC). They demonstrated that *Coca Cola* successfully built a relationship with a senior CDC official, which they leveraged in an attempt to avoid restrictions on sugar-sweetened beverages supported by the *World Health Organization*.

Corporate strategies to stop, delay, or weaken proposed health policies were also frequently discussed. For example, corporations were reported to push for self-regulation schemes [14, 15, 18, 22, 24, 34, 38, 39, 45, 55, 57–72] and to threaten litigation and/or use existing trade treaties to challenge proposed health legislation [23, 71, 73–78]. They were reported to engage in a wide range of argumentative strategies to challenge proposed health policies. For example, corporations suggested that policies were redundant, conflicting, or misaligned with other regulations or international norms [51, 55, 58–60, 73, 79]. Arguments about the impacts of the policy on the economy, employment, equity, and poverty were also common [14, 23, 24, 38, 57–61, 68, 71, 75, 78, 80–83], and economic arguments were considered to be particularly persuasive in low-and-middle income countries (LMICs) [23, 71, 78]. Many articles also discussed the use of front groups as a way to distance the corporation from its unsavoury activities (e.g., lobbying against health policies) [22, 23, 39, 48, 50, 54, 58, 64, 68, 76, 78, 84–86].

#### Preference & perception shaping practices

The ways in which corporations shape preferences for products and influence perceptions about their health-related risks was another major topic of the included articles. Corporations were accused of promoting excessive consumption of harmful products, for example, by engaging in intensive and highly-resourced marketing campaigns that normalize their consumption (e.g., portraying alcohol as part of a normal everyday routine [87]) [14, 18, 22, 38, 43, 48, 53, 57, 64, 68, 76–78, 87–100]. They were reported to influence the public debate about product related-risks by reframing and creating uncertainty about the causes of health issues (e.g., focusing on genetic causes of cancer as opposed to alcohol consumption [101]) [14, 15, 22, 24, 34, 35, 38, 39, 41, 43, 44, 46–48, 50, 52, 54, 56, 57, 57, 58, 62, 63, 69, 79, 80, 84, 102–105] and acquiring or funding media companies, making it more difficult for public health messages to be heard [14, 24, 51, 54, 55, 76]. Though commercial entities promoted education as the solution to managing health-related risks [24, 51, 57, 63, 70, 72, 78, 83], they were also accused of attempting to shape the public’s understanding of health issues by providing educational resources that promoted their products and/or downplayed the associated health risks (e.g., alcohol) [14, 23, 24, 43, 57, 68, 72, 83, 87, 101].

Similarly, commercial actors were accused of promoting products to health professionals by, for example, delivering educational initiatives to health professionals (e.g., the breast-milk substitute (BMS) industry’s funding of medical student retreats [48]) [14, 22–24, 55, 57, 83] and influencing the development of clinical standards (e.g., BMS industry’s funding of clinical guidelines for the diagnosis of cows-milk protein allergy [92]) [54, 82, 92, 98, 106]. Corporations were also reported to influence the academic debate and the production of science by, for example, funding universities, think tanks, and scientific conferences, providing scientific awards, developing industry research institutes and suppressing research that is not aligned with their interests (e.g., failing to publish research on health harms of products) [23, 24, 39, 48, 52–55, 57, 69–72, 76, 79, 83].

#### Corporate Social Responsibility (CSR) practices

CSR initiatives mentioned in the literature include corporate involvement in health promotion programs and contributions to health charities and causes (e.g., tobacco industry’s funding of HIV initiatives [50]; alcohol industry’s involvement in road safety [44]) [22, 23, 34, 44, 45, 48, 50, 57, 71, 76, 92, 98, 107], some of which had limited evidence of effectiveness [22, 34, 45, 50, 76]. Others described CSR initiatives that have implications for health (e.g., diversity, equity, and inclusion efforts) [23, 34, 45, 50, 71, 72]. Corporations were sometimes reported to exploit existing social movements (e.g., women’s rights [66]) to sell products [34, 56, 66] or engage in product reformulation (e.g., ‘light’ cigarettes [54]) to suggest that the company is taking action on product-related harms [18, 22, 43, 57, 72, 93].

Overall, CSR was perceived critically and was seen as being closely related to political practices and preference and perception shaping practices. That is, CSR efforts were seen as attempts to distract from corporate harms [18, 22, 34, 50, 54, 79, 108], shift blame [72, 79], enhance brand image and credibility [23, 43, 50, 71, 87], influence policymaking [23, 44, 50, 54, 72, 75], and/or pre-emptively address threats to business practices [61]. In some cases, these strategic uses of CSR were described by corporations in leaked company documents [54]. Millar [67] suggested that ‘good’ corporations engage in CSR efforts genuinely.

#### Economic practices

Externalization of health harms, for example, through tax avoidance [1, 14, 28, 38, 43, 46, 47, 49, 54, 67, 76, 108–110], was mentioned frequently within the included articles. In this way, corporations were reported to impose health harms onto populations (e.g., chronic disease) without paying for the full cost of these harms. Large monopolies were seen as harmful because they concentrate economic power in one or a few actors, leading to more powerful lobbying efforts [49]. Mendly-Zambo, Raphael, & Taman [107] also discussed the price fixing tactics of *Walmart Canada* and its contributions to food insecurity. Millar [67] described how ‘good’ corporations pay their fair share of taxes and contribute to economic growth.

### Proximal domains of corporate influence

#### Products and services

Several products (e.g., alcohol, tobacco, and gambling machines) were widely recognized as promoting harm. Many authors discussing products referred to their wide availability and accessibility [43, 78, 89, 99, 111, 112]. For example, the ubiquity of fast-food outlets and the provision of free BMS in clinics was seen as increasing consumption of these products [43, 78, 89, 111, 112]. The features of products were also discussed, such as the hyper-palatable nature of ultra-processed foods [94] and the hyper-engagement features of social media (e.g., endless scroll) [69], which were seen as promoting behaviour some authors described as addictive. Liber [113] discussed some health-promoting products (e.g., vaccines) and suggested that regulation should focus on expanding these markets while contracting the markets for health-harming products (e.g., alcohol).

#### Employment practices

Employment practices were not discussed in depth in the included articles. Most articles that discussed employment did so in passing, with reference to health harming practices such as unsafe working conditions, inadequate pay, and limited access to benefits (e.g., parental leave, medical care) [1, 14, 18, 22, 46, 47, 49, 67, 80, 107, 114]. Loewenson [114] provided the deepest discussion of employment by describing the global trend towards precarious labour and associated health effects such as social isolation, high blood pressure, and mental ill-health. Mendly-Zambo, Raphael, and Taman [107] described how *Walmart Canada*’s employment practices (e.g., low wages, anti-union activities) contributed to food insecurity in Canada. In contrast, Millar [67] suggested that ‘good’ businesses create jobs that can have a beneficial effect on human health and described health-promoting activities such as adequate workplace mental health policies.

#### Environmental practices

Health concerns related to environmental practices were also not a significant topic of the included articles. Activities that were mentioned include chemical and pesticide use, air and water pollution, land clearing/deforestation, ecosystem disruption, consumption of energy and water, production of waste, contributions to greenhouse gases, and contributions to biodiversity loss [1, 14, 18, 49, 67, 80]. Kadandale, Marten, and Smith [80] described how slash-and-burn land clearing practices used by the palm oil industry created episodes of harmful haze in South-East Asia, which led to thousands of premature deaths and increases in the rates of respiratory, eye, and skin diseases. Montiel [115] described the health harms of deforestation by pointing to reports that land clearing in Indonesia by the palm oil and sugar industries led to the emergence of the Nipah virus through zoonosis (transfer of a virus from animals to humans).

### Health impact on populations

Guided by the literature, we describe the domains which are relevant to each of the five identified population groups (Table 3). We also describe specific activities relevant to each group that were discussed within the included literature.

**Table 3:** Population Groups Affected by Corporate Activities

Group Whose Health is Impacted	Definition of Group
**Consumers**	Consumers are defined as individuals who buy goods or services produced and/or sold by commercial entities [116]

**Workers**	Workers are defined as individuals who are employed (permanently, contractually, or otherwise) for commercial entities or within their supply chain [117]

**Disadvantaged Groups**	Disadvantaged populations are defined as individuals who are socially, economically, or culturally disadvantaged due to current and/or historical factors (e.g., people of colour, Indigenous populations, migrant populations, women, individuals of low socioeconomic status, citizens of low- and middle-income countries) [118]

**Vulnerable Groups**	Vulnerable groups are defined as groups that are vulnerable due to their age, life stage, or other factors (e.g., children, pregnant persons, older adults, people with disabilities) [25]

**Local Communities**	Local communities are defined as persons living in communities in which corporations are operating (i.e., those affected by physical proximity to corporate operations) [4]

#### Consumers

Consumers, by their nature, are influenced by the properties of consumer goods (products and services). Political and preference and perception shaping practices influence consumers’ health indirectly by creating and shaping the markets for these goods. For example, aggressive marketing can increase the demand for harmful products (e.g., ultra-processed foods) while lobbying and political financing may limit associated protections (e.g., nutrition labelling [55]).

#### Workers

The activities influencing the health of workers are, unsurprisingly, those identified in the employment practices domain. These activities shape the opportunities the worker has to obtain optimal health (e.g., access to medical benefits) and their exposure to harmful or salutogenic factors (e.g., pesticides, positive work environments) [119]. Some political practices (e.g., threatening to shift operations to a country with weaker labour standards) may also impact workers (i.e., through placing downward pressure on labour regulations).

#### Disadvantaged groups

Disadvantaged groups are more likely to be employed in precarious and unsafe jobs [120] and therefore are likely to be strongly affected by employment practices. These groups may also experience the greatest burden from economic practices such as unfair tax practices that reduce funds for social programs. Environmental practices and preference and perception shaping practices are also relevant to these groups [121, 122].

Specific activities described in the literature include disproportionate marketing of unhealthy commodities (e.g., alcohol) to disadvantaged populations [38, 46, 49, 56, 65, 76, 104, 111, 112, 122, 123] and efforts to make unhealthy commodities appealing to women [66, 96, 99]. Millar described increasing domestic income inequities because of large corporate profits which are distributed amongst the elite, who also lobby for reduced taxation [67]. Some private sectors (e.g., the for-profit prison industry, tobacco industry) were accused of placing undue burdens on certain groups (e.g., Black Americans, Indigenous populations) [1, 56, 108, 124–126]. Corporations were reported to push for privatization, which was seen as widening inequities [67, 127]. In some cases, initiatives ostensibly undertaken to improve equity (i.e., efforts to diversify cannabis industry employment) were seen as self-serving attempts to advance industry interests (e.g., increase consumption) [34].

Some articles also described corporate influences on global inequities, including the ‘downward pressure’ on working conditions via corporations’ use of low-wage havens [46, 47, 114, 115], exploitation of the weaker regulatory structures of LMICs, the extraction of wealth from LMICs to HICs [110, 115], and the identification of LMICs as ‘emerging markets’ by unhealthy commodity producers (e.g., tobacco) to replace declines in consumption in HICs [43, 46, 49, 65, 76].

#### Vulnerable groups

The literature described vulnerable groups such as children and pregnant persons being affected by products and associated marketing and educational techniques, as well as the political practices that enable these marketing techniques. Specifically, the included literature discussed food and beverage industry marketing directed towards children and involvement of the industry in schools and other child-centered programming (e.g., distribution of branded school supplies, development of nutrition educational programmes for children) [18, 23, 49, 123]. Pregnant persons and mothers were mostly discussed in the context of the BMS industry and relevant activities included marketing, industry sponsored educational resources, industry interactions with health care professionals, and donations of BMS during emergencies (i.e., ‘crisis marketing’) [22, 24, 48, 53, 77, 78, 92, 128]. Other vulnerable groups (e.g., the elderly or people with disabilities) were not frequently discussed.

#### Local communities

The health outcomes of local communities are likely to be most influenced by environmental and employment practices, especially those occurring in the supply chain, which are enabled by political practices. In the included literature, there was little discussion of specific activities that may influence these communities. One activity that was discussed was the dislocation of local communities [14, 114] through, for example, the activities of the extractive industry [114]. This dislocation was reported to lead to social exclusion, poor access to infrastructure, and dependency on mining activities [114].

## Discussion

In this article, we report on the development of the cross-industry HEALTH-CORP typology. The HEALTH-CORP typology describes 70 corporate activities that can influence population health and health equity, categorized across seven domains of corporate influence. We present a graphical model that situates these domains in relation to their proximity to health outcomes and identify five population groups whose health is influenced by corporate activities.

These work complements and expands on previous work in several ways. Prior typologies of corporate activities tend to be more narrowly focused on either one industry or group of industries [8–10, 13] and/or a specific type of corporate practice (e.g., political practices) [8–12]. To the best of our knowledge, the HEALTH-CORP typology is the most comprehensive typology of health-relevant corporate activities to-date. This novel typology covers a diverse range of corporate activities that were identified based on literature pertaining to 16 different industries. The seven domains we identified are well-aligned with the seven practices (i.e., political practices, scientific practices, marketing practices, supply chain and waste practices, labour and employment practices, financial practices, and reputational management practices) proposed by Gilmore and colleagues [2]; this indicates agreement on the key corporate practices that require our attention within efforts to mitigate the CDH. The activities we identified also share many commonalities with the ‘guiding questions’ provided by Lacy Nichols and colleagues [16], which prompt users (e.g., policy makers) to think about the engagement of commercial entities in activities such as tax avoidance, marketing, lobbying, and providing adequate working conditions. The 70 activities presented in the HEALTH-CORP typology add to this work by more comprehensively identifying the array of different activities through which commercial entities exert influence on population health. Finally, to the best of our knowledge, the HEALTH-CORP model is the first CDH model to include specific population groups that are affected by corporate practices. The identification of these groups may support public health policy and practice, as described later in this discussion.

While the HEALTH-CORP typology adds to prior work, recently published CDH research also suggests ways that the HEALTH-CORP typology could be expanded in the future. For instance, Lacy-Nichols and colleagues ‘guiding questions’ point to topics that the HEALTH-CORP typology could better address in future iterations, such as workplace culture and market consolidation [16]. The HEALTH-CORP typology can be continuously updated as more evidence about the impact of commercial entities on health is identified.

In addition to content, prior work also suggests ways in which the structure of the HEALTH-CORP model and typology could be further developed in the future. For example, the four attributes (i.e., portfolio, resources, organization, and transparency) identified by Lacy Nichols and colleagues [16] may determine the extent to which a company engages in the seven domains identified in the HEALTH-CORP model. The founder of the clothing company *Patagonia*, for example, declined to take the company public because of fear that he would no longer be able to prioritize worker wellbeing and climate action once the company was required to maximize shareholder returns [129]; this example illustrates the potential influence of the company’s organization on its practices. Though it was outside of the scope of this review to consider the influence of these attributes on the identified domains, this is an important area of future inquiry. Likewise, ongoing efforts to identify relationships between specific corporate activities may further elucidate the relationships between the domains identified in the HEALTH-CORP model [15, 29, 35].

The HEALTH-CORP model and typology have several potential uses. The first is to inform policy makers at local, state, national and international levels about the ways that commercial entities can impact population health. The HEALTH-CORP graphic was designed to be relatively simple and easily understandable; it was developed based on principles of infographic design such as the use of simple pictograms and a restricted colour palette [130]. For this reason, it may be an effective visual tool to communicate about corporate practices with policy makers and other stakeholders that may not have an in-depth understanding of the CDH. Moreover, the identification of specific activities and specific population groups affected by these activities may assist policy makers in identifying corporate activities and population groups that are most relevant in their region. For example, public health advocates in low-and middle-income countries may identify workers as a key population group due to the impact that poor employment practices have on workers in these regions (e.g., sweatshops) [131]. Public health actors in other regions may identify certain products (e.g., electronic cigarettes) as priorities based on their impacts on the health of vulnerable populations (e.g., adolescents) [132]. Using the HEALTH-CORP typology as a reference, policy makers could identify which corporate activities may be risk factors (e.g., anti-union activities, marketing) for these health impacts. In the future, the activities in the HEALTH-CORP typology could be mapped to potential policy options to increase its utility to policy makers.

Similarly, the HEALTH-CORP typology and model may be used to inform the work of civil society groups and related social movements that seek to hold corporations to account for their societal impacts. For example, groups such as ShareAction and the *Interfaith Center on Corporate Responsibility* support investors in making socially responsible investments; these organizations also directly engage with companies (e.g., using shareholder resolutions) to encourage them to cease their engagement in health-harming practices [26, 133]. The HEALTH-CORP typology could assist these organizations in identifying the diverse range of corporate activities that have implications for human health. The associated model may also provide these organizations with a visual resource to support their engagement with companies and investors.

Finally, the HEALTH-CORP typology and model could be used to support efforts to measure and monitor corporate activities that impact population health and health equity, a key priority for the CDH field [4, 8, 13]. Activities within the HEALTH-CORP typology (e.g., lobbying) could be transformed into specific indicators (e.g., annual amount spent on lobbying, issues lobbied) that allow for comparisons of commercial practices within and across industries and regions. This could allow CDH scholars as well as national and local governments to track harmful corporate practices, assess their impact on specific health outcomes, and identify priorities for regulatory action. Though there are existing surveillance and benchmarking efforts dedicated to tracking corporate practices, these efforts often focus on a particular industry and/or health issue (e.g., the Access to Nutrition Index) or do not focus specifically on human health (e.g., the World Benchmarking Alliance) [2, 25, 134]. The HEALTH-CORP typology may provide the conceptual infrastructure for cross-industry monitoring efforts that capture the diverse range of commercial activities that affect population health.

Finally, our review and synthesis of a substantial number of CDH articles allowed us to identify two key research gaps in the CDH literature. First, the included literature reported extensively on political and preference and perception shaping practices, providing countless examples of how these practices have been manifested in different scenarios. However, some of the proximal domains (i.e., employment practices, environmental practices) were explored only superficially. This gap may be limiting our ability to understand the complex interactions between different types of corporate practices and may also limit our ability to determine which corporate activities are most health-harming and therefore should be prioritized for intervention. To address this gap, CDH scholars could engage in collaborative research partnerships with environmental and occupational health experts. A first step may be to connect with the researchers involved in the *Lancet* Countdown on health and climate change and the authors from the *Lancet* ‘Work and Health’ series [119, 135]. Authors of the latter series have called for stronger monitoring of indicators of workers’ health that capture the social and environmental determinants of health (e.g., psychosocial risk factors within the workplace) [136]. Likewise, the existing Global Burden of Disease Study allows for the identification of occupational and environmental risk factors that correspond to high burdens of mortality and morbidity (e.g., long working hours) [137]. These risk factor estimates may point to important economic features (e.g., the gig economy) and practices (e.g., working-time arrangements) precipitating these risk factors that should be further explored using a CDH lens [138].

Another gap that we identified based on the findings of our review is the limited investigation of the ways in which commercial entities influence the health of disadvantaged groups, vulnerable groups, and local communities. This may, in part, reflect the relative lack of CDH research on LMICs compared to HICs [3]. Understanding the impact of corporate practices on these communities will be paramount to addressing domestic and global health inequities. Efforts to advance our understanding of these activities may be facilitated by the Corporate Health Impact Assessment tool developed by Baum and colleagues [4], which can be used to conduct in-depth investigations of commercial entities within regions of interest. Researchers may also consider conducting community-based participatory research [139] with communities of interest to better understand the corporate activities that most affect them and determine how individuals in these communities weigh positive impacts of corporate involvement in their community (e.g., employment) against negative impacts (e.g., water pollution).

### Limitations

We were able to review and synthesize a substantial number of articles (116) on the CDH across 16 industries to develop a typology that describes corporate activities that can influence health across multiple domains. We believe the HEALTH-CORP typology will be useful for advancing scholarship, policy, and practice related to the CDH. The typology nor the associated model, however, can completely explain the complex system through which commercial entities influence health. CDH researchers could seek to further refine and/or add to the activities in the typology as well as investigate the structures (e.g., type of ownership), features (e.g., norms), and other actors (e.g., governments) that play a role in driving these activities. Likewise, the five population groups that we identified are not likely to be all of the populations affected by commercial activities; additional groups could be identified in future work. Moreover, though we included all activities mentioned within the included articles that could be reasonably linked to human health in the HEALTH-CORP typology; it was beyond the scope of this review to assess the strength of evidence of the relationship of each activity to health or the magnitude of the associated health burden. Since most of the included literature described corporate practices from a negative perspective, the HEALTH-CORP typology is primarily focused on activities that lead to health harms. The typology could be improved in future iterations by more fully considering the benefits some companies provide to society (e.g., inexpensive goods manufactured efficiently at large-scales).

Our eligibility criteria did not include grey and non-English literature which may have improved the comprehensiveness of the HEALTH-CORP typology. Importantly, we also limited our review to articles that directly engaged with CDH terms, thereby excluding research that may describe corporate practices that influence health without engaging with CDH concepts. We made this decision for feasibility purposes and because this strategy allowed us to identify key gaps in CDH literature, a contribution of this review. Finally, the constant comparative method used to build the typology was conducted by one author only. Other authors contributed to the refinement of the typology and associated model at later stages of development.

## Conclusions

Scholarship on the CDH has documented the wide-ranging influence that corporate activities can have on population health. In this article, we make three contributions to the CDH literature. First, we present the HEALTH-CORP typology, which describes 70 corporate activities that can influence population health across industries and categorizes these activities into seven domains of corporate influence. Second, we provide a graphical model that situates the domains based on their proximity to health outcomes and describes five population groups to consider when evaluating the health impact of corporate practices. Finally, we leverage our findings to reveal key gaps in CDH literature and recommend future avenues for CDH research. We believe these contributions will be useful for advancing public health research and practice related to the CDH.

## Supplementary Information

Supplementary Material 1.

## Data Availability

The data for this study are published academic articles which are available from the respective publishers (see Appendix 3 for the characteristics of included articles). In addition, we uploaded the following files to Open Science Framework (10.17605/OSF.IO/TG9S7) to support data availability: 1) a.csv file containing a list of the articles that underwent title and abstract screening in our study and the respective screening decisions that were assigned, and 2).ris files containing the citations to the respective articles and the assigned screening decisions, which can be uploaded into a reference manager. Interested parties can contact the corresponding author for additional information.

## Abbreviations

CDH: Commercial determinants of health
SDH: Social determinants of health
HEALTH-CORP: Corporate Influences on Population Health
PRISMA-ScR: PRISMA Extension for Scoping Reviews
CSR: Corporate Social Responsibility
CDC: Centers for Disease Control and Prevention
HICs: High-income countries
LMICs: Low- and middle-income countries
F&B: Food and beverage
