# Supplementary material for: Corporate activities that influence population health: a scoping review and qualitative synthesis to develop the HEALTH-CORP typology
Source: Global Health. 2024 Nov 9;20:77. doi: 10.1186/s12992-024-01082-4 (PMC11549802; doi:10.1186/s12992-024-01082-4)
Supplement: Supplementary file 1 — Supplementary Material 1. [file 12992_2024_1082_MOESM1_ESM.docx]

**Additional File**

**Table of Contents**

[Appendix 1: Search Strategy Used in Review 2](#_Toc172627579)

[Appendix 2: PRISMA Diagram 9](#_Toc172627580)

[Appendix 3: Table Describing the Characteristics of Included Articles 10](#_Toc172627581)

[Appendix 4: The Corporate Influences on Health (HEALTH-CORP) Typology 17](#_Toc172627582)

[Appendix 5: Activities Recorded in One Industry (Not Included in Typology) 22](#_Toc172627583)

[Appendix 6: Industries in Which the Domains of Influence Were Discussed 23](#_Toc172627584)

# Appendix 1: Search Strategy Used in Review

**Databases:** Scopus, OVID Medline, Ovid Embase, Ovid Global Health

Searched from inception on Sept 13, 2022

| Database | Search String |
| --- | --- |
| Scopus | TITLE-ABS-KEY ((( corporat* OR commercial*) AND determinant* ) OR "corporate political activity" OR "corporate political activities") AND ( health* OR disease* OR wellbeing OR "well being" OR morbidit* OR mortalit* OR "life expectanc* " OR DALY OR DALYs OR "disability-adjusted life year" OR "disability-adjusted life years" OR QALY OR QALYs OR "quality-adjusted life year" OR "quality-adjusted life years") |
| OVID Medline(R) | ((( corporat* OR commercial*) AND determinant*) OR "corporate political activity" OR "corporate political activities") AND ( health* OR disease* OR wellbeing OR "well being" OR morbidit* OR mortalit* OR "life expectanc*" OR DALY OR DALYs OR "disability-adjusted life year" OR "disability-adjusted life years" OR QALY OR QALYs OR "quality-adjusted life year" OR "quality-adjusted life years").mp. [mp=title, abstract, original title, name of substance word, subject heading word, floating sub-heading word, keyword heading word, organism supplementary concept word, protocol supplementary concept word, rare disease supplementary concept word, unique identifier, synonyms]. |
| Embase | (((corporat* OR commercial*) AND determinant*) OR "corporate political activity" OR "corporate political activities") AND ( health* OR disease* OR wellbeing OR "well being" OR morbidit* OR mortalit* OR "life expectanc*" OR DALY OR DALYs OR "disability-adjusted life year" OR "disability-adjusted life years" OR QALY OR QALYs OR "quality-adjusted life year" OR "quality-adjusted life years").mp. [mp=title, abstract, heading word, drug trade name, original title, device manufacturer, drug manufacturer, device trade name, keyword heading word, floating subheading word, candidate term word] |
| Global Health | ((( corporat* OR commercial*) AND determinant*) OR "corporate political activity" OR "corporate political activities") AND ( health* OR disease* OR wellbeing OR "well being" OR morbidit* OR mortalit* OR "life expectanc*" OR DALY OR DALYs OR "disability-adjusted life year" OR "disability-adjusted life years" OR QALY OR QALYs OR "quality-adjusted life year" OR "quality-adjusted life years").mp. [mp=abstract, title, original title, broad terms, heading words, identifiers, cabicodes] |

# Appendix 2: PRISMA Diagram

# Appendix 3: Table Describing the Characteristics of Included Articles

| Author(s) (Year) | Title | Type of Article | Industry/ies | Regions | Research-Specific Funding Reported? (Y/N) | Type of Funder | Reported Conflict of Interest? (Y/N)^a^ | Open-Access?  (Y/N) |
| --- | --- | --- | --- | --- | --- | --- | --- | --- |
| Millar, J. (2013) | The Corporate Determinants of Health: How Big Business Affects Our Health, and the Need for Government Action! | Commentary | General, with focus on food and beverage | Canada | N | - | N | Y |
| Kickbusch, I. Allen, L., Franz, C. (2016) | The commercial determinants of health | Commentary | General | - | N | - | N | Y |
| Buse, K., Tanaka, S., Hawkes, S (2017) | ﻿Healthy people and healthy profits? Elaborating a conceptual framework for governing the commercial determinants of non-communicable diseases and identifying options for reducing risk exposure | Conceptual | Tobacco, ultra-processed foods, alcohol | Global | Y | Educational Institution | N | Y |
| Knai, C. et al. (2018) | Systems Thinking as a Framework for Analyzing Commercial Determinants of Health | Conceptual | General | - | Y | Philanthropic | N | Y |
| McKee, M, Stuckler, D. (2018) | Revisiting the corporate and commercial determinants of health | Conceptual | General | - | N | - | N | Y |
| Wiist, W. (2019) | Mechanisms Underlying Corporations as Determinants of Health | Response | General, focus on tobacco | - | N | - | Y | Y |
| Brown, T. (2019) | Legislative capture: A critical consideration in the commercial determinants of public health | Conceptual | General, with focus on alcohol | Australia | N | - | N | N |
| Ireland, R., Chambers, S., Bunn, C. (2019) | Exploring the relationship between Big Food corporations and professional sports clubs: a scoping review | Review | Food and beverage | Global | Y | Educational Institution | N | Y |
| Toebes, B.,  Patterson, D. (2019) | Human rights and Non-Communicable Diseases: Controlling Tobacco and Promoting Healthy Diets (Book Chapter) | Conceptual | General, focus on tobacco and food and beverage | Global | N | - | N | N |
| Hessari, N. et al. (2019) | Recruiting the “heavy-using loyalists of tomorrow”: An analysis of the aims, effects and mechanisms of alcohol advertising, based on advertising industry evaluations | Qualitative | Alcohol | United Kingdom | Y | Educational Institution | N | Y |
| Peres, M et al. (2019) | Oral diseases: a global public health challenge | Conceptual | Food and beverage | Global | N | - | N | Y |
| Battams, S. & Townsend, B. (2019) | Power asymmetries, policy incoherence and noncommunicable disease control - a qualitative study of policy actor views | Qualitative | General, focus on tobacco, alcohol, food and beverage | Global, Switzerland, Australia, Malaysia | Y | Educational Institution | N | N |
| Madureira Lima, J. & Galea, S. (2019) | The Corporate Permeation Index – A tool to study the macrosocial determinants of Non-Communicable Disease | Scale Development | General | Global | N | - | N | Y |
| Hessari, N. et al. (2019) | Public Meets Private: Conversations Between Coca-Cola and the CDC | Qualitative | Beverage | United States | Y | Philanthropic | Y | Y |
| Ireland, R. et al. (2019) | Commercial determinants of health: Advertising of alcohol and unhealthy foods during sporting events | Conceptual | Alcohol, food and beverage | United Kingdom | Y | Government Entity | Y | Y |
| Kadandale, S., Marten, R., Smith, R. (2019) | The palm oil industry and noncommunicable diseases | Conceptual | Food (palm oil) | Global | N | - | N | Y |
| Kasture, A et al. (2019) | Benchmarking the commitments related to population nutrition and obesity prevention of major food companies in New Zealand | Scale Application | Food and beverage | New Zealand | N | - | N | Y |
| Fooks, G. et al. (2019) | Corporations' use and misuse of evidence to influence health policy: A case study of sugar-sweetened beverage taxation | Qualitative | Beverage | South Africa | N | - | N | Y |
| Kenworthy, N. (2019) | Crowdfunding and global health disparities: an exploratory conceptual and empirical analysis | Conceptual | Crowdfunding | Global | Y | Educational Institution | N | Y |
| Rochford, C., Tenneti, N., Moodie, R. (2019) | Reframing the impact of business on health: the interface of corporate, commercial, political and social determinants of health | Commentary | General | - | N | - | N | Y |
| Mialon et al. (2020) | ‘We must have a sufficient level of profitability’: food industry submissions to the French parliamentary inquiry on industrial food | Qualitative | Food & beverage | France | Y | Government entity | N | N |
| Eastmure, E., Cummins, S., Sparks, L. (2020) | Non-market strategy as a framework for exploring commercial involvement in health policy: A primer | Conceptual | General |  | N |  | N | Y |
| Maani, N., Abdalla, S., Galea, S. (2020) | The firearm industry as a commercial determinant of health | Commentary | Firearm | United States | N | - | N | N |
| Lacy-Nicholas, J., Scrinis, G., Carey, R. (2020) | The politics of voluntary self-regulation: Insights from the development and promotion of the Australian Beverages Council's Commitment | Qualitative | Beverage | Australia | N | - | N | Y |
| Brown, T (2020) | Public health vs alcohol industry compliance laws: A case of regulatory capture? | Conceptual | Alcohol | Australia | N | - | N | N |
| Sacks, G et al. (2020) | Benchmarking the nutrition-related policies and commitments of major food companies in Australia, 2018 | Scale Application | Food and beverage | Australia | N | - | Y | Y |
| Hastings et al. (2020) | Selling second best: how infant formula marketing works | Qualitative | Baby food | United Kingdom, Continental Europe, North America, Australia and New Zealand | Y | International Governance Organization | N | Y |
| De Lacy-Vawdon, C. & Livingstone, C. (2020) | Defining the commercial determinants of health: a systematic review | Review | General | - | N | - | N | Y |
| Mialon, M., Crosbie, E., Sacks, G. (2020) | Mapping of food industry strategies to influence public health policy, research and practice in South Africa | Qualitative | Food and beverage | South Africa | Y | Philanthropic | N | Y |
| Mialon, M. et al. (2020) | Arguments used by trade associations during the early development of a new front-of-pack nutrition labelling system in Brazil | Qualitative | Food and beverage | Brazil | Y | Government Entity | N | N |
| Hill, S., Friel, S. (2020) | ‘As Long as It Comes off as a Cigarette Ad, Not a Civil Rights Message’: Gender, Inequality and the Commercial Determinants of Health | Conceptual | Tobacco, alcohol |  | N |  | N | Y |
| Mialon, M. (2020) | An overview of the commercial determinants of health | Review | General | - | N | - | N | Y |
| Tanrikulu, H. et al. (2020) | Corporate political activity of the baby food industry: The example of Nestlé in the United States of America | Qualitative | Baby food | United States | N | - | N | Y |
| Hoe, C. et al. (2020) | Drink, but don't drive? The alcohol industry's involvement in global road safety | Qualitative | Alcohol | Global | Y | International Governance Organization | N | Y |
| Mialon, M. (2020) | Food industry political practices in Chile: “the economy has always been the main concern” | Qualitative | Food and beverage | Chile | Y | Educational Institution; Government Entity | N | Y |
| Lauber et al., (2020) | Non-communicable disease governance in the era of the sustainable development goals: A qualitative analysis of food industry framing in WHO consultations | Qualitative | Food and beverage | Global | N | - | N | Y |
| Jamieson, L., Gibson, B., Thomson, W. (2020) | Oral health inequalities and the corporate determinants of health: A commentary | Commentary | Food and beverage, alcohol, tobacco | - | N | - | N | Y |
| Karim, Kruger & Hofman (2020) | Industry strategies in the parliamentary process of adopting a sugar-sweetened beverage tax in South Africa: a systematic mapping | Qualitative | Beverage | South Africa | Y | Government entity | N | Y |
| Mialon et al. (2020) | “The architecture of the state was transformed in favour of the interests of companies”: corporate political activity of the food industry in Colombia | Qualitative | Food and beverage | Colombia | Y | Educational Institution; Government Entity | N | Y |
| Petticrew et al. (2020) | Dark Nudges and Sludge in Big Alcohol: Behavioral Economics, Cognitive Biases, and Alcohol Industry Corporate Social Responsibility | Qualitative | Alcohol | - | N | - | N | Y |
| McCarthy et al. (2020) | ‘It’s a tradition to go down to the pokies on your 18th birthday’ – the normalisation of gambling for young women in Australia | Qualitative | Gambling | Australia | Y | Government Entity | N | Y |
| Howse, E et al. (2021) | Air pollution and the noncommunicable disease prevention agenda: Opportunities for public health and environmental science | Commentary | General | - | N | - | N | Y |
| van Schalkwyk, M et al. (2021) | A public health approach to gambling regulation: countering powerful influences | Commentary | Gambling | United Kingdom | N | - | Y | Y |
| Cossez, E., Baker, P., Mialon, M. (2021) | ‘The second mother’: How the baby food industry captures science, health professions and civil society in France | Qualitative | Baby food | France | N | - | N | Y |
| Madden, M. & McCambridge, J. (2021) | Alcohol marketing versus public health: David and Goliath? | Commentary | Alcohol | Global | N | - | N | Y |
| Fisher, L et al. (2021) | Barriers and opportunities to restricting marketing of unhealthy foods and beverages to children in Nepal: a policy analysis | Qualitative | Food and beverage | Nepal | Y | Educational Institution; Government Entity | N | Y |
| Stubbs, T. (2021) | Commercial determinants of youth smoking in ASEAN countries: A narrative review of research investigating the influence of tobacco advertising, promotion, and sponsorship | Review | Tobacco | ASEAN (Association of Southeast Asian Nations) | Y | Educational Institution | N | Y |
| Mialon, M et al. (2021) | Beyond nutrition and physical activity: food industry shaping of the very principles of scientific integrity | Qualitative | General, food and beverage | - | Y | Philanthropic | N | Y |
| Wood, B., Baker, P., Sacks, G. (2021) | Conceptualising the Commercial Determinants of Health Using a Power Lens: A Review and Synthesis of Existing Frameworks | Review | General | - | N | - | N | Y |
| Chavez-Ugalde, Y et al. (2021) | Conceptualizing the commercial determinants of dietary behaviors associated with obesity: A systematic review using principles from critical interpretative synthesis | Review | Food and beverage | - | N | - | N | Y |
| Lauber, K et al. (2021) | Corporate political activity in the context of unhealthy food advertising restrictions across Transport for London: A qualitative case study | Qualitative | Food and beverage | United Kingdom | N | - | Y | Y |
| Freudenberg, N et al. (2021) | Defining Priorities for Action and Research on the Commercial Determinants of Health: A Conceptual Review | Review | General | - | Y | Government entity | N | Y |
| Zenone, M et al. (2021) | How does the British Soft Drink Association respond to media research reporting on the health consequences of sugary drinks? | Qualitative | Beverage | United Kingdom | N | - | N | Y |
| Milaon, M et al. (2021) | Involvement of the food industry in nutrition conferences in Latin America and the Caribbean | Qualitative | Food and beverage | Latin America, Caribbean | N | - | Y | Y |
| Hunt, D. (2021) | How food companies use social media to influence policy debates: A framework of Australian ultra-processed food industry Twitter data | Qualitative | Food and beverage | Australia | N | - | N | Y |
| Wood, B et al. (2021) | Market strategies used by processed food manufacturers to increase and consolidate their power: a systematic review and document analysis | Review | Food and beverage | - | N | - | N | Y |
| Wood, B et al. (2021) | The double burden of maldistribution: a descriptive analysis of corporate wealth and income distribution in four unhealthy commodity industries | Quantitative | Food and beverage, Alcohol, Tobacco, Fossil Fuels | Global, USA | N | - | N | N |
| Lacy-Nichols, J. & Marten, R. (2021) | Power and the commercial determinants of health: Ideas for a research agenda | Commentary | General | - | N | - | N | Y |
| Zenone, M. & Kenworthy, N. (2021) | Pre-emption strategies to block taxes on sugar-sweetened beverages: A framing analysis of Facebook advertising in support of Washington state initiative-1634 | Qualitative | Beverage | United States | N | - | N | Y |
| Loewenson, R. (2021) | Rethinking the Paradigm and Practice of Occupational Health in a World Without Decent Work: A Perspective From East and Southern Africa | Commentary | General, Mining | East and Southern Africa | N | - | N | Y |
| Knai, C et al. (2021) | The case for developing a cohesive systems approach to research across unhealthy commodity industries | Workshop | General | - | Y | Philanthropic | N | Y |
| Mendly-Zambo, Z., Raphael, D., Taman, A. (2021) | Take the money and run: how food banks became complicit with Walmart Canada’s hunger producing employment practices | Conceptual | Retail | Canada | N | - | N | N |
| Hyder, A et al. (2021) | The COVID-19 Pandemic Exposes Another Commercial Determinant of Health: The Global Firearm Industry | Commentary | Firearm | - | Y | Educational Institution | N | Y |
| Campbell, N et al. (2021) | The Gift of Data: Industry-Led Food Reformulation and the Obesity Crisis in Europe | Qualitative | Food and beverage | United Kingdom, Portugal, Ireland, Germany, France | N | - | N | N |
| Zenone, M., Kenworthy, N., Barbic, S. (2021) | The Paradoxical Relationship Between Health Promotion and the Social Media Industry | Commentary | Social media | - | N | - | Y | Y |
| Gerritsen, S et al. (2021) | The Timing, Nature and Extent of Social Media Marketing by Unhealthy Food and Drinks Brands During the COVID-19 Pandemic in New Zealand | Qualitative | Food and beverage | New Zealand | Y | Philanthropic | N | Y |
| Klein, D. & Lima, J. (2021) | The Prison Industrial Complex as a Commercial Determinant of Health | Commentary | Prison | United States | N | - | N | Y |
| McHardy, J. (2021) | The WHO FCTC's lessons for addressing the commercial determinants of health | Commentary | General, tobacco | Global | N | - | Y | Y |
| Russ, K et al. (2021) | What You Don’t Know About the Codex Can Hurt You: How Trade Policy Trumps Global Health Governance in Infant and Young Child Nutrition | Mixed methods | Baby food | Global | Y | Philanthropic | Y | Y |
| Baker, P et al. (2021) | Breastfeeding, first-food systems and corporate power: a case study on the market and political practices of the transnational baby food industry and public health resistance in the Philippines | Qualitative | Baby food | Philippines | Y | International Governance Organization | Y | Y |
| Baker, P et al. (2021) | First-food systems transformations and the ultra-processing of infant and young child diets: The determinants, dynamics and consequences of the global rise in commercial milk formula consumption | Review | Baby food | Global | Y | International Governance Organization | N | Y |
| Baker, P et al. (2021) | Globalization, first-foods systems transformations and corporate power: a synthesis of literature and data on the market and political practices of the transnational baby food industry | Review | Baby food | Global | Y | International Governance Organization | Y | Y |
| Jones et al. (2021) | Disrupting the commercial determinants of health; Chapter 5 in “Australia in 2030 – What is our path to health for all?” (Supplement) | Conceptual | General | Australia | Y | Government Entity | N | Y |
| Maani et al. (2021) | The new WHO Foundation – global health deserves better | Commentary | General | Global | N | - | Y | Y |
| Maani et al. (2021) | The need for a conceptual understanding of the macro and meso commercial determinants of health inequalities | Commentary | General, Alcohol, Tobacco | - | N | - | N | Y |
| Brisbois et al. (2021) | Mining, colonial legacies, and neoliberalism: A political ecology of health knowledge | Conceptual | Extractive | Canada | N | - | Y | Y |
| Jia et al. (2021) | #SupportLocal: how online food delivery services leveraged the COVID-19 pandemic to promote food and beverages on Instagram | Mixed Methods | Food and beverages | Australia, New Zealand, United Kingdom, United States, Canada | N | - | N | Y |
| Mialon et al. (2021) | ‘I had never seen so many lobbyists’: food industry political practices during the development of a new nutrition front-of-pack labelling system in Colombia | Qualitative | Food and beverage | Colombia | Y | Educational Institution; Government Entity | N | Y |
| Dall’Alba & Rocha (2021) | Brazil’s response to COVID-19: commercial determinants of health and regional inequities matter | Commentary | General | Brazil | N | - | N | Y |
| Diderichsen et al. (2021) | Beyond ‘commercial determinants’: shining a light on privatization and political drivers of health inequalities | Commentary | General | Sweden, Denmark | N | - | N | Y |
| Yates et al. (2021) | Trust and responsibility in food systems transformation. Engaging with Big Food: marriage or mirage? | Conceptual | Food and beverage | Global | N | - | N | Y |
| Maani et al. (2021) | The Commercial Determinants of Three Contemporary National Crises: How Corporate Practices Intersect With the  COVID-19 Pandemic, Economic Downturn, and Racial Inequity | Commentary | General | United States | N | - | N | Y |
| Van Schalkwyk et al. (2021) | Our Postpandemic World: What Will It Take to Build a Better Future for People and Planet? | Commentary | General | Global | N | - | N | Y |
| Adams, Rychert, & Wilkins (2021) | Policy inﬂuence and the legalized cannabis industry: learnings from other addictive consumption industries | Conceptual | Cannabis | New Zealand | N | - | Y | N |
| Boatwright et al. (2021) | The Politics of Regulating Foods for Infants and Young Children: A Case Study on the Framing and Contestation of Codex Standard-Setting Processes on Breast-Milk Substitutes | Mixed Methods | Baby food | Global | Y | International Governance Organization | N | Y |
| Watts, Burton & Freman (2021) | ‘The last line of marketing’: Covert tobacco marketing tactics as revealed by former tobacco industry employees | Qualitative | Tobacco | Australia | Y | Philanthropic | N | Y |
| Gillespie et al. (2021) | Conceptualising changes to tobacco and alcohol policy as affecting a single interlinked system | Workshop | Tobacco, alcohol | United Kingdom | Y | Philanthropic; Government Entity; Educational Institution | N | Y |
| Jamieson et al. (2021) | Neoliberalism and Indigenous oral health inequalities: a global perspective | Commentary | Tobacco, food and beverage | Global | N | - | N | N |
| Hill et al. (2021) | From silos to policy coherence: tobacco control, unhealthy commodity industries and the commercial determinants of health | Commentary | Tobacco, General | - | N | - | Y | Y |
| McCarthy, S et al. (2022) | Electronic gambling machine harm in older women: a public health determinants perspective | Qualitative | Gambling | Australia | N | - | Y | N |
| Lee, K et al. (2022) | Measuring the Commercial Determinants of Health and Disease: A Proposed Framework | Scale Development | General | - | Y | Government Entity | N | Y |
| Allen, Wigley, and Homer (2022) | Assessing the association between Corporate Financial Influence and implementation of policies to tackle commercial determinants of non-communicable diseases: A cross-sectional analysis of 172 countries | Quantitative | General | Global | N | - | N | Y |
| Buse, Mialon, Jones (2022) | Thinking politically about UN political declarations: A recipe for healthier commitments – free of commercial interests | Conceptual | General | - | N | - | N | Y |
| Clare, Maani, and Milner (2022) | Meat, money and messaging: How the environmental and health harms of red and processed meat consumption are framed by the meat industry | Qualitative | Food (meat) | United Kingdom | N | - | N | N |
| Barlow & Allen (2022) | The impact of trade and investment agreements on the implementation noncommunicable disease policies, 2014-2019: protocol for a statistical study (preprint) | Quantitative Protocol | Tobacco, alcohol, food and beverage | Global | N | - | N | Y |
| DuPont-Reyes, Hernandez-Munoz, and Tang (2022) | TV advertising, corporate power, and Latino health disparities | Mixed Methods | Alcohol, tobacco, food and beverage, pharmaceutical | United States | N | - | N | N |
| Gokani et al. (2022) | UK Nutrition Research Partnership ‘Hot Topic’ workshop report: A ‘game changer’ for dietary health – addressing the implications of sport sponsorship by food businesses through an innovative interdisciplinary collaboration | Workshop | Food and beverage | United Kingdom | Y | Government Entity | N | Y |
| Hird et al. (2022) | Understanding the long-term policy influence strategies for the tobacco industry: two contemporary case studies | Conceptual | Tobacco | - | Y | Philanthropic | N | Y |
| Montiel et al. (2022) | Tracing the connections between international business and communicable diseases | Conceptual | General | Global | Y | Educational Institution | N | Y |
| Fooks & Godziewski (2022) | The World Health Organization, Corporate Power, and the Prevention and Management of Conflicts of Interest in Nutrition Policy: Comment on “Towards Preventing and Managing Conflict of Interest in Nutrition Policy? An Analysis of Submissions to a Consultation on a Draft WHO Tool” | Commentary | Food and beverage | Global | N | - | N | Y |
| Zenone, Kenworthy, & Maani (2022) | The Social Media Industry as a Commercial Determinant of Health | Commentary | Social media |  | N | - | N | Y |
| Mialon et al. (2022) | Conflicts of interest for members of the U.S. 2020 Dietary Guidelines Advisory Committee | Quantitative | Food and beverage | United States | Y | Philanthropic | N | Y |
| Passini et al. (2022) | Conflict of interests in the scientific production of Vitamin D and COVID-19: A Scoping Review | Review | Food, diagnostics, pharmaceutical | - | Y | Educational Institution | N | Y |
| Ramsbottom et al. (2022) | Food as harm reduction during a drinking session: reducing the harm or normalising harmful use of alcohol? A qualitative comparative analysis of alcohol industry and non-alcohol industry-funded guidance | Qualitative | Alcohol | - | N | - | N | Y |
| Rose, Reeve & Charlton (2022) | Barriers and Enablers for Healthy Food Systems and Environments: The Role of Local Governments | Review | Food and beverage | - | N | - | N | Y |
| Steele et al. (2022) | Confronting potential food industry ‘front groups’: case study of the international food information Council’s nutrition communications using the UCSF food industry documents archive | Qualitative | Food and beverage | Global | Y | Philanthropic | Y | Y |
| De Lacy-Vawdon, Vandenberg, & Livingstone (2022) | Recognising the elephant in the room: the commercial determinants of health | Commentary | General | - | N | - | N | Y |
| Wiist, W. (2022) | The Foundations of Corporate Strategies: Comment on “‘Part of the Solution’: Food Corporation Strategies for Regulatory Capture and Legitimacy” | Commentary | General | United States | N | - | Y | Y |
| Maani et al. (2022) | Manufacturing doubt: Assessing the effects of independent vs industry-sponsored messaging about the harms of fossil fuels, smoking, alcohol, and sugar sweetened beverages | Quantitative | Alcohol, tobacco, fossil fuels, sugar-sweetened beverages | United Kingdom | Y | Government Entity | N | Y |
| Hoe et al. (2022) | Strategies to expand corporate autonomy by the tobacco, alcohol and sugar-sweetened beverage industry: a scoping review of reviews | Review | Tobacco, alcohol, sugar-sweetened beverages | - | Y | Philanthropic | N | Y |
| Freudenberg (2022) | Responding to Food Industry Initiatives to Be “Part of the Solution”  Comment on “‘Part of the Solution’: Food Corporation Strategies for Regulatory Capture and Legitimacy” | Response | Food and beverage | - | N | - | N | Y |
| Kroker-Lobos et al. (2022) | Two countries, similar practices: The political practices of the food industry influencing the adoption of key public health nutrition policies in Guatemala and Panama | Qualitative | Food | Guatemala, Panama | Y | Educational Institution; Government Entity | N | Y |
| Leimbigler et al. (2022) | Social, political, commercial, and corporate determinants of rural health equity in Canada: an integrated framework | Commentary | General | Canada | Y | Educational Institution | N | Y |
| Liber (2022) | Using Regulatory Stances to See All the Commercial Determinants of Health | Conceptual | General, Pharma/Diagnostics, Food & beverage, Housing, Tobacco (E-Cigarettes) | - | N | - | Y | Y |
| Wakefield, Glantz & Appollonio (2022) | Content Analysis of the Corporate Social Responsibility Practices of 9 Major Cannabis Companies in Canada and the US | Qualitative | Cannabis | United States, Canada | Y | Government Entity | N | Y |
| Van Schalkwyk, Hawkins & Pettigrew (2022) | The politics and fantasy of the gambling education discourse: An analysis of gambling industry-funded youth education programmes in the United Kingdom | Qualitative | Gambling | United Kingdom | Y | Government Entity | N | Y |

^a^Articles labelled with ‘N’ in this column either specifically declared that there was no related funding, reported funding that was not specific to the research (e.g., general support for investigators) or information on funding was not available (i.e., not reported). ‘Y’ indicated funding specific to the article in question was reported.

^b^Articles labelled with ‘N’ in this column either specifically declared no competing interests or information on competing interests was not available (i.e., not declared). ‘Y’ indicates that a competing interest was declared.

# Appendix 4: The Corporate Influences on Health (HEALTH-CORP) Typology

| Domains of Corporate Influence | Definition of Domain | Corporate Activities with Potential to Influence Population Health and/or Health Equity^a,b^ | Expected Direction of Health Impact^c^ |
| --- | --- | --- | --- |
| DISTAL DOMAINS | | | |
| Political Practices  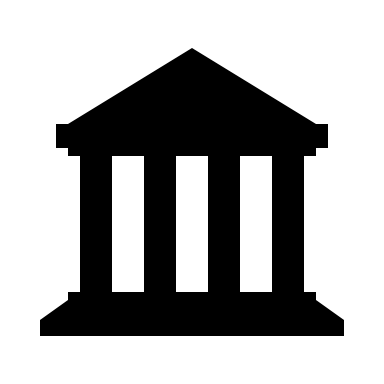 | This domain consists of activities undertaken to influence government policy or processes in ways that are favourable to the commercial entity [1]. | ***Activities related to securing a favourable policy environment:*** | |
|  |  | Engage in political financing | Depends |
|  |  | Engage in bribery (e.g., provide gifts or other incentives to policy makers) | - |
|  |  | Advocate for policies that limit corporate liability for health harms (e.g., right to refuse surprise inspections by regulatory agencies [1]) | - |
|  |  | Engage in efforts to develop relationships between corporations and public health institutions (e.g., through public-private partnerships, funding, ‘wine-ing and dining’) or other relevant groups (e.g., minority rights groups, patient groups) and associated persons | Depends |
|  |  | Advocate for the placement of corporate representatives on regulatory boards | - |
|  |  | Exploit the use of ‘revolving doors’ (i.e., employees who move between positions in industry and government [2]) | - |
|  |  | Take or threaten legal action in response to unfavourable policies | - |
|  |  | Advocate for engagement in regulation (e.g., self-regulation, co-regulation, or voluntary codes) | - |
|  |  | Engage in political lobbying, including through the use of third parties (e.g., front groups) | Depends |
|  |  | Engage in strategies (e.g., introducing ballot measures [3]) to leverage pre-emption (i.e., higher levels of government restrict the jurisdiction of lower levels [4]) or venue-shifting (i.e., the transfer of policy-making to a jurisdiction that may be more likely to advance the company’s interests [5]) | Depends |
|  |  | Leverage trade treaties to challenge unfavourable policies | - |
|  |  | Amplify influence via front groups or industry alliances/coalitions | - |
|  |  | Use argumentative strategies to oppose and/or delay proposed health policies  (e.g., suggest policy is not within the mandate of the regulating institution [6]) [5] | - |
|  |  | Misrepresent evidence or demand unrealistic standards of public health evidence within policy submission processes | - |
|  |  | Exploit existing divisions in the public health community or otherwise attempt to weaken opposing groups (e.g., through supporting multiple bills to deflect attention from their primary legislative target [7]) | - |
|  |  | Engage in intimidation tactics, including efforts to discredit opposing scientists and policy makers | - |
|  |  | Shift or threaten to shift operations to countries with weaker regulations | - |
|  |  | ***Other political activities:*** |  |
|  |  | Advocate for privatization of public services | Depends |
|  |  | Expropriate land (i.e., land grabs) for industry activities (e.g., mining [8]) | - |
| Preference & Perception Shaping Practices  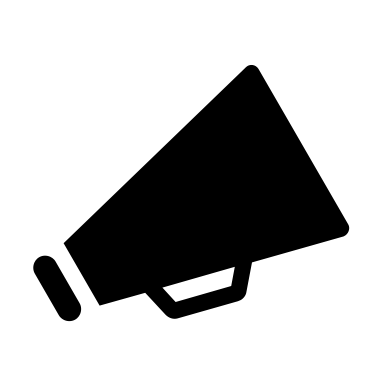 | This domain consists of activities that shape preferences for products and/or influence perceptions about products and their health-related harms [1]. | ***Activities related to promoting products:*** | |
|  |  | Engage in marketing to encourage product recognition and consumption | Depends |
|  |  | Engage in marketing of harmful products in ways that disproportionately targets disadvantaged or vulnerable groups (e.g., placing more advertisements on certain platforms [9], ‘feminizing’ harmful commodities [10], using flavours to appeal to youth [11]) | - |
|  |  | Leverage pandemics (e.g., COVID-19 [12]) or disasters to promote consumption of harmful products (sometimes referred to as ‘crisis marketing’ [13]) | - |
|  |  | Sponsor sports teams, music, or other cultural events, relevant individuals (e.g., professional athletes, celebrities, social media influencers), and/or related infrastructure (e.g., sporting arenas) | Depends |
|  |  | ***Activities related to shaping the public debate about products & their health implications:*** | |
|  |  | Engage in health education efforts directed at the public (e.g., alcohol industry’s messaging about the health harms of alcohol consumption during pregnancy [14]) | Depends |
|  |  | Conduct educational and/or advocacy campaigns to influence the public’s perception of health policies (e.g., spread misinformation about policies on social media [3]) | - |
|  |  | Craft and/or propagate inaccurate or skewed narratives about the causes of health issues (e.g., suggest that physical activity is more important than diet in weight management [15]), including through the use of front groups | - |
|  |  | Advance the ideas of individual responsibility for health and consumer choice | Depends |
|  |  | Acquire ownership, establish relationships, or exert influence on the media via spending on advertisements [1,16] | - |
|  |  | Employ medical experts, scientists, or other key opinion leaders to advance industry interests (e.g., by giving lectures supporting harmful products [17], writing position papers, or holding industry-sponsored workshops [1,18]) | - |
|  |  | ***Activities related to shaping the professional debate about products & their health implications:*** | |
|  |  | Provide funding to professional associations | Depends |
|  |  | Contribute to the development of clinical standards (e.g., cows-milk protein allergy [19]) | Depends |
|  |  | Engage in health education efforts targeted at health care professionals (or health care professional students) | Depends |
|  |  | ***Activities related to the production of evidence and the academic debate about products and their health implications:*** | |
|  |  | Provide funding for research in ways that can shapes both the direction (e.g., funding is provided for certain industry-friendly topics over others [20]) and research outcomes (e.g., findings and conclusions [21]). Funding may be provided to scientists and research groups/institutes, universities, scientific conferences, academic journals, scientific awards, or think tanks | Depends |
|  |  | Suppress, amplify, or cherry-pick research depending on its desirability to industry (e.g., disseminate industry-favourable research social media bots [22]) | - |
|  |  | Obscure conflicts of interest (i.e., relationships between authors and industry [23]) in research (e.g., through ghost writing [24]) | - |
|  |  | Contribute to the development of scientific standards (e.g., principles of scientific integrity [25], standards of scientific ‘proof’ [26]) | Depends |
|  |  | Falsify or misrepresent data | - |
| Corporate Social Responsibility Practices 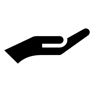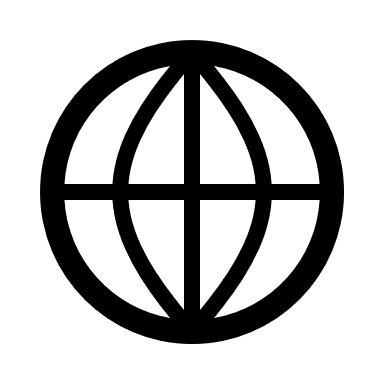 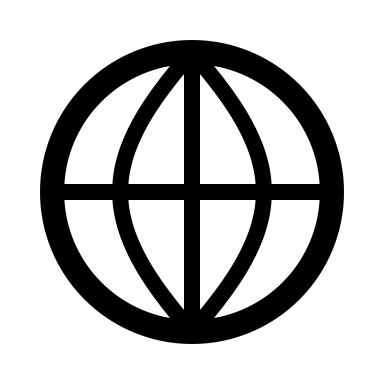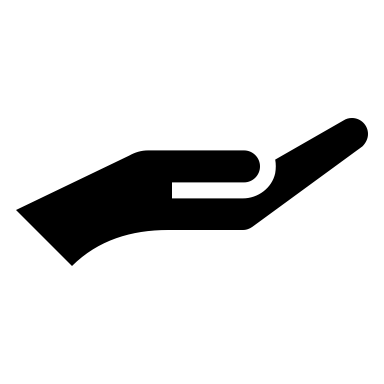 | This domain consists activities undertaken with the stated intention to contribute to society and/or offset environmental, social, or health impacts of previous activities [27]. | Develop, shape, or contribute resources to health promotion programs (e.g., programs to reduce drunk driving [28]), health charities and non-profit organizations (e.g., HIV prevention initiatives [17]) | Depends |
|  |  | Engage in other social responsibility initiatives that are relevant to health (e.g., diversity, equity, and inclusion efforts [27]) | Depends |
|  |  | Engage with existing social movements (e.g., the women’s rights movement [29]) | Depends |
|  |  | Reformulate products for health-related reasons (e.g., develop drinks with artificial sweeteners to reduce sugar content [30]) | Depends |
| Economic Practices  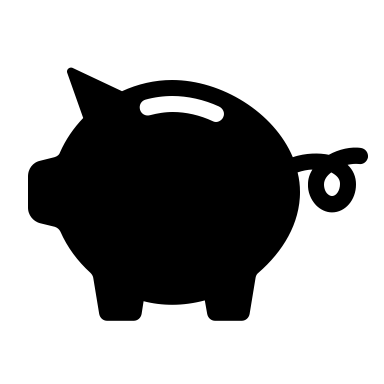 | This domain consists of activities that influence the economy and the distribution of wealth within society [31]. | Engage in fair or unfair tax practices (e.g., tax evasion, tax avoidance [32]) | +/- |
|  |  | Engage in profiteering or price fixing of necessary commodities (e.g., food [33]) | - |
|  |  | Contribute to economic growth and related benefits (e.g., improvements in infrastructure, education, healthcare) | + |
|  |  | Contribute to inequitable distributions of wealth and power through ownership and renumeration structures that prioritize the accrual of wealth to certain individuals (i.e., executives, shareholders) over others (i.e., workers) | - |
| PROXIMAL DOMAINS | | | |
| Products & Services  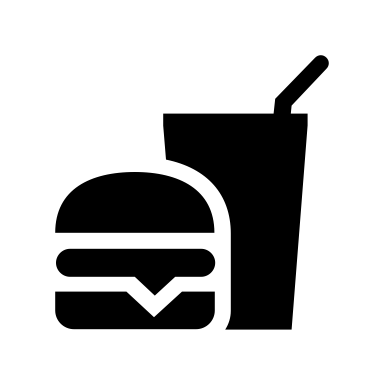 | This domain consists of activities related to the production and sale of products and services [34]. | ***Activities related to the characteristics of products:*** | |
|  |  | Develop, produce, or sell products with harmful (e.g., cigarettes) or salutogenic properties (e.g., vaccines) or those that are essential to human life (e.g., food) | +/- |
|  |  | Develop, produce, or sell products with addictive properties | +/- |
|  |  | ***Activities related to the accessibility of products:*** | |
|  |  | Determine the price of products (for e.g., low prices of ultra-processed foods, use of price promotions, discounts, coupons, offers, and vouchers [35]) | Depends |
|  |  | Determine the physical proximity and availability of products to consumers (e.g., hours of operation of gambling outlets [36]) | Depends |
| Employment Practices  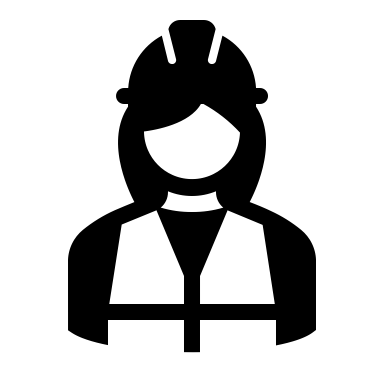 | This domain consists of activities related to the conditions under which employment is provided [37]. | ***Activties related to determining the characteristics of employment:*** | |
|  |  | Determine the number of employment opportunities, their type (e.g., skill level), and geographic distribution | Depends |
|  |  | Determine the adequacy of pay in relation to local living standards | +/- |
|  |  | Determine the stability of employment terms | +/- |
|  |  | ***Activities related to the benefits received through employment:*** | |
|  |  | Determine the provision and quality of medical benefits | +/- |
|  |  | Determine the provision and quality of pension plans | +/- |
|  |  | Determine the provision, length, and paid or unpaid status of employee leave (e.g., parental, personal) | +/- |
|  |  | Determine the provision and quality of employee wellness programs | +/- |
|  |  | ***Activities related to the conditions of employment:*** |  |
|  |  | Determine the quality of working conditions (physical and psychosocial) | +/- |
|  |  | Determine the extent to which workers are free to engage in unionization or collective bargaining without interference or fear of reprisal | +/- |
|  |  | Determine the provision of support for breastfeeding in the workplace (e.g., availability of lactation rooms [38]) | +/- |
|  |  | Determine the provision of opportunities to work remotely and the characteristics of remote work (e.g., organizational support [8]) | +/- |
|  |  | ***Other employment-related activities:*** | |
|  |  | Determine the presence of child labor or forced labor directly or in the supply chain | +/- |
| Environmental Practices  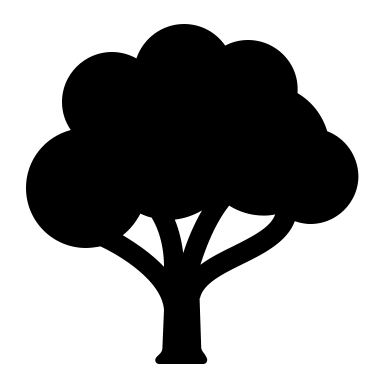 | This domain consists of corporate activities that can influence physical and/or mental health through the impact of the activity on the natural environment [39]. | Use or avoid the use of harmful chemicals and pesticides | +/- |
|  |  | Determine contributions to air pollution, including efforts to prevent air pollution | +/- |
|  |  | Determine contributions to water pollution, including efforts to prevent water pollution | +/- |
|  |  | Determine the production of waste, including efforts to reduce, reuse, and recycle | +/- |
|  |  | Determine the extent of resource extraction (e.g., water), including efforts to conserve resources | +/- |
|  |  | Determine contributions to deforestation, including efforts to prevent deforestation and to reforest | +/- |
|  |  | Determine the consumption and conservation of energy, including the source (e.g., renewable) | +/- |
|  |  | Determine the extent of greenhouse gas emissions, including efforts to reduce emissions | +/- |
|  |  | Determine contributions to the loss of biodiversity, including efforts to prevent loss and engage in ecosystem restoration | +/- |

^a^The corporate activities are described using verbs (e.g., contribute, engage) to draw attention to the active role that corporations play in shaping population health. However, we recognize that there are other actors (e.g., the government) involved that play a role in how these activities are enacted (e.g., federal regulations for parental leave policies).

^b^The expected associated health impact may be direct (such as producing health-harming products) or indirect (such as using argumentative strategies to oppose proposed health policies within policy submissions).

^c^A “+” indicates that the respective activity is expected to have a positive impact on human health. A “-” indicates that the respective activity is expected to have a negative impact on health. A “+/-” indicates that the activity can produce a positive or a negative impact on human health depending on the extent to which the activity is present or absent or its quality. For example, providing medical benefits to employees would likely result in a positive health impact whereas not doing so would likely result in a negative health impact [40]. A “Depends” indicates that whether the impact on human health is positive or negative depends on the specifics of the respective activity. For example, providing funding for a scientific conference may have an indirect positive or negative impact on human health *depending* on whether the funding allows the corporation to influence the agenda of the respective conference. The direction of expected health impact was identified based on the included literature as well as by consulting additional literature sources as needed. For example, through product reformulation (e.g., reducing salt in food products) could be considered a health-promoting activity, the expected direction of impact was recorded as ‘Depends’ because the included literature described how product reformulation could be used as a mechanism to suggest the industry is taking action independently, with the effect of preventing stronger, government-endorsed restrictions on product contents [41].

# Appendix 5: Activities Recorded in One Industry (Not Included in Typology)

The following activities were recorded with respect to one industry only and therefore were not included in the HEALTH-CORP typology:

| Corporate Activities with Potential to Influence Population Health and/or Health Equity | Direction of Expected Health Impact^a^ | Industry in Which the Activity Was Discussed |
| --- | --- | --- |
| Determine the presence of socially-isolated employee communities (e.g., migrant or ‘fly-in’ workers) | +/- | Extractive |
| Determine the provision and quality of harm prevention supports | +/- | Gambling |
| Place restrictions on corporate data (making it difficult to assess health harms) | - | Social Media |
| Undermine social safety nets | - | Crowdfunding |
| Engage in unapproved research | - | Food & Beverage |

^a^A “+” indicates that the respective activity is expected to have a positive impact on human health. A “-” indicates that the respective activity is expected to have a negative impact on health. A “+/-” indicates that the activity can produce a positive or a negative impact on human health depending on the extent to which the activity is present or absent or its quality. For example, providing medical benefits to employees would likely result in a positive health impact whereas not doing so would likely result in a negative health impact [40]. A “Depends” indicates that whether the impact on human health is positive or negative depends on the specifics of the respective activity. For example, providing funding for a scientific conference may have an indirect positive or negative impact on human health *depending* on whether the funding allows the corporation to influence the agenda of the respective conference.

# Appendix 6: Industries in Which the Domains of Influence Were Discussed

| Domains of Corporate Influence | General | Food & Beverage | Tobacco | Alcohol | Baby Food | Gambling | Extractive | Pharma and Diagnostics | Firearms | Social Media | Cannabis | Prisons | Retail | Crowd-funding | E-Cigarettes | Housing | Fossil Fuels | Total number of industries identified per domain |
| --- | --- | --- | --- | --- | --- | --- | --- | --- | --- | --- | --- | --- | --- | --- | --- | --- | --- | --- |
| Political Practices |  |  |  |  |  |  |  |  |  |  |  |  |  |  |  |  |  | **9** |
| Preference & Perception Shaping Practices |  |  |  |  |  |  |  |  |  |  |  |  |  |  |  |  |  | **11** |
| Corporate Social Responsibility (CSR) Practices |  |  |  |  |  |  |  |  |  |  |  |  |  |  |  |  |  | **10** |
| Economic Practices |  |  |  |  |  |  |  |  |  |  |  |  |  |  |  |  |  | **7** |
| Products & Services |  |  |  |  |  |  |  |  |  |  |  |  |  |  |  |  |  | **12** |
| Employment Practices |  |  |  |  |  |  |  |  |  |  |  |  |  |  |  |  |  | **5** |
| Environmental Practices |  |  |  |  |  |  |  |  |  |  |  |  |  |  |  |  |  | **3** |
| Total number of domains identified for respective industry | **7** | **7** | **5** | **4** | **6** | **4** | **5** | **3** | **2** | **2** | **2** | **1** | **4** | **1** |  | **1** | **2** | **-** |

**References in Additional File**

 1. Madureira Lima J, Galea S. Corporate practices and health: A framework and mechanisms. Globalization and Health. 2018;14:1–12.

2. Definition of “the revolving door” [Internet]. Collins Dictionary. Glasgow, UK: HarperCollins; Available from: https://dictionary.cambridge.org/dictionary/english/consumer

3. Zenone M, Kenworthy N. Pre-emption strategies to block taxes on sugar-sweetened beverages: A framing analysis of Facebook advertising in support of Washington state initiative-1634. Global Public Health. 2021;

4. Crosbie E, Schillinger D, Schmidt LA. State Preemption to Prevent Local Taxation of Sugar-Sweetened Beverages. JAMA Intern Med. 2019;179:291.

5. Ulucanlar S, Lauber K, Fabbri A, Hawkins B, Mialon M, Hancock L, et al. Corporate Political Activity: Taxonomies and Model of Corporate Influence on Public Policy. 2023 [cited 2023 May 1]; Available from: https://dx.doi.org/10.34172/ijhpm.2023.7292

6. Lauber K, Ralston R, Mialon M, Carriedo A, Gilmore AB. Non-communicable disease governance in the era of the sustainable development goals: A qualitative analysis of food industry framing in WHO consultations. Globalization and Health. 2020;16.

7. Ulucanlar S, Fooks GJ, Gilmore AB. The Policy Dystopia Model: An Interpretive Analysis of Tobacco Industry Political Activity. PLoS medicine. 2016;13:e1002125.

8. Loewenson R. Rethinking the Paradigm and Practice of Occupational Health in a World Without Decent Work: A Perspective From East and Southern Africa. New Solutions. 2021;31:107–12.

9. DuPont-Reyes MJ, Hernandez-Munoz JJ, Tang L. TV Advertising, Corporate Power, and Latino Health Disparities. American Journal of Preventive Medicine. 2022;63:496–504.

10. Atkinson AM, Meadows BR, Emslie C, Lyons A, Sumnall HR. ‘Pretty in Pink’ and ‘Girl Power’: An analysis of the targeting and representation of women in alcohol brand marketing on Facebook and Instagram. International Journal of Drug Policy. 2022;101:103547.

11. Mosher JF, Johnsson D. Flavored Alcoholic Beverages: An International Marketing Campaign that Targets Youth. J Public Health Pol. 2005;26:326–42.

12. Gerritsen S, Sing F, Lin K, Martino F, Backholer K, Culpin A, et al. The Timing, Nature and Extent of Social Media Marketing by Unhealthy Food and Drinks Brands During the COVID-19 Pandemic in New Zealand. Frontiers in Nutrition. 2021;8.

13. Baker P, Zambrano P, Mathisen R, Singh-Vergeire MR, Escober AE, Mialon M, et al. Breastfeeding, first-food systems and corporate power: a case study on the market and political practices of the transnational baby food industry and public health resistance in the Philippines. Globalization and Health. 2021;17.

14. Maani N, van Schalkwyk MCI, Filippidis FT, Knai C, Petticrew M. Manufacturing doubt: Assessing the effects of independent vs industry-sponsored messaging about the harms of fossil fuels, smoking, alcohol, and sugar sweetened beverages. SSM - Population Health. 2022;17.

15. Buse K, Tanaka S, Hawkes S. Healthy people and healthy profits? Elaborating a conceptual framework for governing the commercial determinants of non-communicable diseases and identifying options for reducing risk exposure. Globalization and Health. 2017;13:34.

16. Bohme SR, Zorabedian J, Egilman DS. Maximizing Profit and Endangering Health: Corporate Strategies to Avoid Litigation and Regulation. International Journal of Occupational and Environmental Health. 2005;11:338–48.

17. Adams PJ, Rychert M, Wilkins C. Policy influence and the legalized cannabis industry: learnings from other addictive consumption industries. Addiction. 2021;116:2939–46.

18. Sismondo S. Key Opinion Leaders and the Corruption of Medical Knowledge: What the Sunshine Act Will and Won’t Cast Light On. The Journal of Law, Medicine & Ethics. 2013;41:635–43.

19. Baker P, Santos T, Neves PA, Machado P, Smith J, Piwoz E, et al. First-food systems transformations and the ultra-processing of infant and young child diets: The determinants, dynamics and consequences of the global rise in commercial milk formula consumption. Maternal and Child Nutrition. 2021;17.

20. Fabbri A, Lai A, Grundy Q, Bero LA. The Influence of Industry Sponsorship on the Research Agenda: A Scoping Review. https://doi.org/102105/AJPH2018304677. 2018;108:e9–16.

21. Lundh A, Lexchin J, Mintzes B, Schroll JB, Bero L. Industry sponsorship and research outcome: systematic review with meta-analysis. Intensive Care Med. 2018;44:1603–12.

22. Steele S, Sarcevic L, Ruskin G, Stuckler D. Confronting potential food industry ‘front groups’: case study of the international food information Council’s nutrition communications using the UCSF food industry documents archive. Globalization and Health. 2022;18.

23. Thompson DF. Understanding Financial Conflicts of Interest. N Engl J Med. 1993;329:573–6.

24. Savell E, Gilmore AB, Fooks G. How Does the Tobacco Industry Attempt to Influence Marketing Regulations? A Systematic Review. PLOS ONE. 2014;9:e87389.

25. Mialon M, Ho M, Carriedo A, Ruskin G, Crosbie E. Beyond nutrition and physical activity: food industry shaping of the very principles of scientific integrity. Globalization and Health. 2021;17.

26. Legg T, Hatchard J, Gilmore AB. The Science for Profit Model—How and why corporations influence science and the use of science in policy and practice. PLOS ONE. 2021;16:e0253272.

27. Wakefield T, Glantz SA, Apollonio DE. Content Analysis of the Corporate Social Responsibility Practices of 9 Major Cannabis Companies in Canada and the US. JAMA Network Open. 2022;E2228088.

28. Hoe C, Taber N, Champagne S, Bachani AM. Drink, but don’t drive? The alcohol industry’s involvement in global road safety. Health policy and planning. 2021;35:1328–38.

29. Hill SE, Friel S. ‘As long as it comes off as a cigarette ad, not a civil rights message’: Gender, inequality and the commercial determinants of health. International Journal of Environmental Research and Public Health. 2020;17:1–19.

30. Mialon M, Corvalan C, Cediel G, Scagliusi FBB, Reyes M. Food industry political practices in Chile: “the economy has always been the main concern.” Globalization and Health. 2020;16.

31. Baum FE, Sanders DM, Fisher M, Anaf J, Freudenberg N, Friel S, et al. Assessing the health impact of transnational corporations: Its importance and a framework. Globalization and Health. 2016;12:1–7.

32. Wood B, McCoy D, Baker P, Williams O, Sacks G. The double burden of maldistribution: a descriptive analysis of corporate wealth and income distribution in four unhealthy commodity industries. Critical Public Health. 2021;

33. Mendly-Zambo Z, Raphael D, Taman A. Take the money and run: how food banks became complicit with Walmart Canada’s hunger producing employment practices. Critical Public Health. 2023;33:60–71.

34. Knai C, Petticrew M, Capewell S, Cassidy R, Collin J, Cummins S, et al. The case for developing a cohesive systems approach to research across unhealthy commodity industries. BMJ global health. 2021;6.

35. Chavez-Ugalde Y, Jago R, Toumpakari Z, Egan M, Cummins S, White M, et al. Conceptualizing the commercial determinants of dietary behaviors associated with obesity: A systematic review using principles from critical interpretative synthesis. Obesity Science and Practice. 2021;7:473–86.

36. McCarthy S, Thomas S, Pitt H, Daube M, Cassidy R. ‘It’s a tradition to go down to the pokies on your 18th birthday’ – the normalisation of gambling for young women in Australia. Australian and New Zealand Journal of Public Health. 2020;44:376–81.

37. Occupational Safety and Health [Internet]. International Labour Organization. 2024 [cited 2024 Aug 14]. Available from: https://libguides.ilo.org/occupational-safety-and-health-en/home

38. Kim JH, Shin JC, Donovan SM. Effectiveness of Workplace Lactation Interventions on Breastfeeding Outcomes in the United States: An Updated Systematic Review. Journal of Human Lactation. 2019;35:100–13.

39. Sattler B. Environmental Health. Policy, Politics, & Nursing Practice. 2003;4:4–5.

40. Van Niel MS, Bhatia R, Riano NS, De Faria L, Catapano-Friedman L, Ravven S, et al. The Impact of Paid Maternity Leave on the Mental and Physical Health of Mothers and Children: A Review of the Literature and Policy Implications. Harvard Review of Psychiatry. 2020;28:113–26.

41. Hoe C, Weiger C, Minosa MKR, Alonso F, Koon AD, Cohen JE. Strategies to expand corporate autonomy by the tobacco, alcohol and sugar-sweetened beverage industry: a scoping review of reviews. Globalization and Health. 2022;18:17.
